# Non-coding RNAs as key players in neurodegeneration and brain tumors: Insights into therapeutic strategies

**DOI:** 10.22038/ijbms.2025.85350.18446

**Published:** 2025

**Authors:** Mohamed Javad Saadh, Sarraa Ahmad Qahtan, Rafid Jihad Albadr, Gaurav Sanghvi, Rangaswamy Roopashree, Aditya Kashyap, A. Sabarivani, Jasur Rizaev, Waam Mohammed Taher, Mariem Alwan, Mahmod Jasem Jawad, Ali M. Ali Al-Nuaimi

**Affiliations:** 1 Faculty of Pharmacy, Middle East University, Amman, 11831, Jordan; 2 Department of Anesthesia Techniques, Health and Medical Techniques College, Alnoor University, Mosul, Iraq; 3 Ahl al Bayt University, Karbala, Iraq; 4 Marwadi University Research Center, Department of Microbiology, Faculty of Science, Marwadi University, Rajkot-360003, Gujarat, India; 5 Department of Chemistry and Biochemistry, School of Sciences, JAIN (Deemed to be University), Bangalore, Karnataka, India; 6 Centre for Research Impact & Outcome, Chitkara University Institute of Engineering and Technology, Chitkara University, Rajpura, 140401, Punjab, India; 7 Department of Biomedical, Sathyabama Institute of Science and Technology, Chennai, Tamil Nadu, India; 8 Department of Public Health and Healthcare Management, Rector, Samarkand State Medical University, 18, Amir Temur Street, Samarkand, Uzbekistan; 9 College of Nursing, National University of Science and Technology, Dhi Qar, Iraq; 10 Pharmacy college, Al-Farahidi University, Iraq; 11 Department of Pharmacy, Al-Zahrawi University College, Karbala, Iraq; 12 Gilgamesh Ahliya University, Baghdad, Iraq

**Keywords:** Alzheimer’s disease, Brain cancer, Extracellular vesicles Glioma, Long non-coding RNA, MicroRNA Neurodegenerative diseases, Non-coding RNA

## Abstract

Non-coding RNAs (ncRNAs), including microRNAs (miRNAs), long non-coding RNAs (lncRNAs), and other ncRNA types, have emerged as key regulators in neurodegenerative diseases and brain tumors. This review aims to provide insights into the role of ncRNAs in these conditions and their potential as diagnostic and therapeutic targets. We systematically reviewed literature from databases such as PubMed, Scopus, and Web of Science, applying specific inclusion and exclusion criteria to ensure comprehensive coverage of recent advancements. Although ncRNAs are involved in a range of molecular pathways, challenges in clinical translation, including specificity, cost, and validation, persist. This review highlights innovative strategies to overcome these barriers and promote the clinical application of ncRNAs. Moreover, we explore the emerging role of extracellular vesicle-enriched ncRNAs as cell-free therapeutic options for neurodegenerative diseases. The findings presented here emphasize the need for robust validation and the development of specific ncRNA-based treatments.

## Introduction

The whole range of RNA molecules expressed in a cell or tissue is referred to as the transcriptome, making up over 90% of the human genome, while less than 2% of genes encode proteins. Most transcribed genes produce non-coding RNAs (ncRNAs), which have been classified into different types (1, 2). However, the classification is not well established due to the excessive similarity between ncRNAs and protein-coding RNAs in terms of transcriptional and post-transcriptional behavior, the length of ncRNAs, and the composition of the nucleotide sequence (3).

Length of ncRNAs is a factor in categorizing them into short (19-31 nucleotides), mid (20-200 nucleotides), and long (>200 nucleotides) RNAs. Small ncRNAs (sncRNAs) comprising ribosomal RNA (rRNA), transfer RNA (tRNA), microRNA (miR), piwi-interacting RNA (piRNA), and small nucleolar RNA (snoRNA) are crucial gene expression regulators (4, 5). Among the mentioned classes, miRs and long ncRNAs (lncRNAs) are the most considerably studied ones, degrading messenger RNA (mRNA) and hindering translation through imperfect or near-perfect base pairing and acting as guidance for chromatin modulation complexes or nucleolar transcription factors, respectively (6).

Recent research has highlighted the critical role of ncRNAs in both neurodegeneration and brain tumor progression. NcRNAs, including miRNAs, lncRNAs, and others, regulate key biological processes involved in disease progression, such as gene expression modulation, apoptosis, inflammation, and cell cycle regulation. These ncRNAs influence several signaling pathways crucial for neuronal survival and tumorigenesis. For example, miRNAs modulate pathways like PI3K/Akt, MAPK, Wnt/β-catenin, and autophagy (7, 8), which are involved in cell survival, inflammation, and oncogenesis. Furthermore, lncRNAs have been implicated in regulating tumor suppressors and oncogenes, thereby influencing tumor progression and resistance to treatment (9).

Neuron loss coinciding with irreversible tissue lesions is generally defined as central nervous system (CNS) disorders or neurodegenerative diseases presenting dramatic phenotypic and behavioral changes (10). Compelling evidence has shown that various ncRNAs are involved in human disease processes (11). The central nervous system (CNS) has the most elevated profusion, specificity, and interactions of ncRNAs, which suggest cognitive evolution in mammals. Furthermore, dysregulations of ncRNAs, such as over-aggregation/abundance, may affect neurodegeneration, which has also been reported in various neurological disorders, including Alzheimer’s disease (AD), Parkinson’s disease (PD), and Huntington’s disease (HD) (12-14). According to the World Health Organization (WHO), AD affects over 55 million people worldwide, with an estimated 10 million new cases annually. PD, the second most common neurodegenerative disorder, affects approximately 10 million individuals globally, with increasing prevalence as populations age. These conditions are characterized by progressive neuronal degeneration, which leads to irreversible cognitive decline, motor dysfunction, and ultimately, death (15).

Anomalous brain cells may grow uncontrollably, resulting in brain tumors, causing about 250,000 deaths in 2020, emphasizing the pivotal timely diagnosis and therapy for modification of the survival rates, which have been about 36% and 31% for the 5-year and 10-year survival rates, respectively (16). There is now considerable evidence pointing towards ncRNAs associating with various disease processes, including cancer (17). In addition, modulated gene expressions implicated in tumorigenesis, tumor stage progression, and metastasis have been associated with deviant ncRNA expression in cancers, such as pituitary adenoma and glioma, which are the most common primary brain tumors of the CNS (18-20).

**Figure 1:**
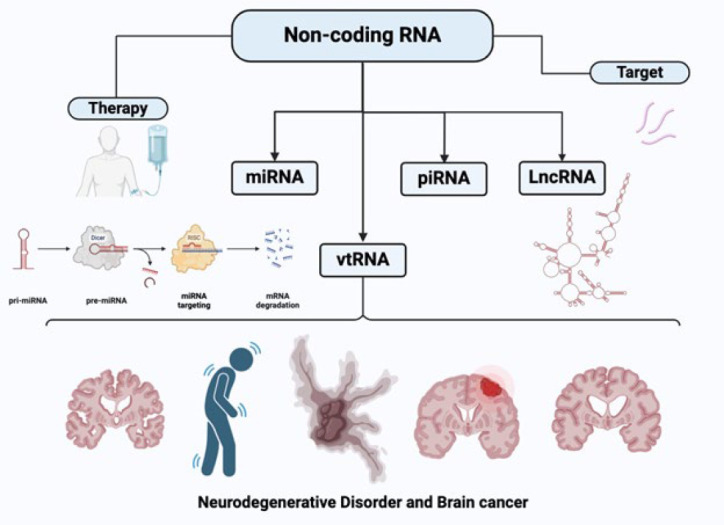
The graphical abstract investigation

ncRNAs deliver substantial benefits as a therapeutic target, as their small molecular weight varies from macromolecular antibody drugs, allowing them to penetrate the tissue barrier and reach the tumor’s interior more efficiently. Therefore, a profound impact on the future therapeutics against diverse diseases will be gained through recent studies, resulting in advancements in genome-wide transcriptomic investigations, which will aid in reassuring therapeutics for future generations (21, 22). This review is the first to comprehensively examine ncRNAs’ roles in neurodegenerative diseases and brain tumors, providing a unique perspective on their shared mechanisms and potential therapeutic targets. While previous studies have focused on ncRNAs in either neurodegeneration or cancer individually, our review highlights how these molecules simultaneously contribute to the pathology of both conditions, offering new insights for future therapeutic strategies. We systematically reviewed the literature from several databases, including PubMed, Scopus, and Web of Science, using predefined inclusion and exclusion criteria to select relevant studies. This review aims to integrate findings from multiple studies and provide a comprehensive understanding of the ncRNA-mediated mechanisms involved in both brain tumors and neurodegeneration (Figure 1).

## Materials and Methods

To ensure a comprehensive and systematic review of the literature on ncRNAs in neurodegeneration and brain tumors, we employed a rigorous methodology for selecting and including studies. We conducted a systematic search of major scientific databases, including PubMed, Scopus, and Web of Science, using combinations of keywords such as “non-coding RNA,” “ncRNA,” “microRNA,” “miRNA,” “long non-coding RNA,” “lncRNA,” “piwi-interacting RNA,” “piRNA,” “vault RNA,” “extracellular vesicles,” “exosomes,” “neurodegeneration,” “neurodegenerative diseases,” “Alzheimer’s disease,” “Parkinson’s disease,” “Huntington’s disease,” “amyotrophic lateral sclerosis,” “brain tumors,” “glioma,” “glioblastoma,” “therapeutic strategies,” and “diagnostic biomarkers.” Studies were included if they focused on the role of ncRNAs in neurodegenerative diseases or brain tumors, provided significant insights into molecular mechanisms, diagnostic potential, or therapeutic applications, and were published in English within the last 10 years. However, seminal earlier works were also considered. Studies were excluded if they were irrelevant to the topic, lacked methodological rigor, were published in non-English languages, or were redundant with previously included studies (23, 24).

The literature search yielded numerous potential studies, which were managed through a two-step screening process. In the initial screening, titles and abstracts were reviewed to determine relevance, and studies that did not meet the inclusion criteria were excluded. The remaining studies underwent a full-text review to assess their suitability based on the inclusion and exclusion criteria, ensuring that only high-quality, relevant studies were included. For each included study, relevant data were extracted, including study details (authors, publication year, study design, and objectives), the specific ncRNA(s) studied, the disease context, key findings related to ncRNA roles in disease mechanisms, diagnostic potential, or therapeutic applications, and the experimental methods employed. The quality of the studies was assessed based on methodological rigor, reproducibility, and the significance of the findings in advancing the understanding of ncRNAs in neurodegeneration and brain tumors. The extracted data were synthesized to provide a comprehensive overview of the current knowledge of ncRNAs in neurodegeneration and brain tumors, highlighting key themes such as their role in disease mechanisms, potential as diagnostic biomarkers, and therapeutic applications. While every effort was made to ensure a comprehensive and unbiased review, some limitations should be acknowledged, including possible publication bias towards studies with positive results, excluding non-English studies, and the possibility of missing relevant studies not indexed in the searched databases. Based on the gaps identified in the literature, we recommend that future research focus on clinical validation of ncRNA biomarkers, preclinical testing of EV-based therapies, and developing standardized protocols for EV isolation and ncRNA analysis to improve reproducibility and reliability. By adhering to this rigorous methodology, we aimed to provide a comprehensive and unbiased review of the current state of knowledge on ncRNAs in neurodegeneration and brain tumors while identifying key areas for future research (25).

### Overview of ncRNAs

**Figure 2:**
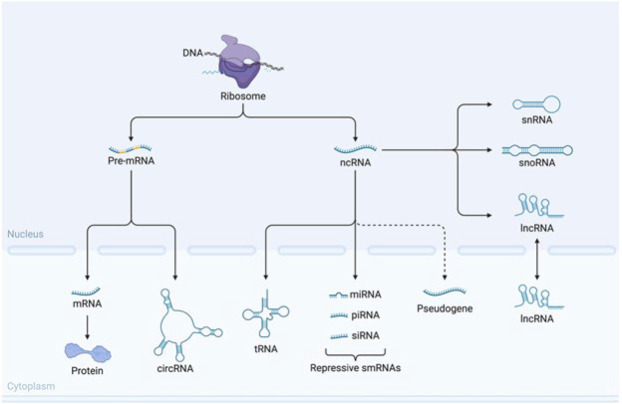
The diagram illustrates molecular biology’s central dogma, showing DNA’s transcription and processing into various RNA types, followed by their functions in the cytoplasm

ncRNAs are RNA molecules that do not code for proteins, which can be divided into two main categories: lncRNAs and sncRNAs. LncRNAs are typically longer than 200 nucleotides and have regulatory roles in gene expression. In comparison, sncRNAs are usually shorter than 200 nucleotides. They can act as regulators of gene expression, as well as having other functions in the cell, such as ribosome biogenesis and mRNA stability. ncRNAs have been found to play vital roles in various biological processes such as development, stem cell differentiation, immune response, metabolism, and cancer (26, 27). Research has demonstrated that lncRNA HOXA11-AS exacerbates neuronal damage induced by MPTP in SH-SY5Y cells and contributes to the activation of microglia by lipopolysaccharide (LPS), promoting an increased proportion of pro-inflammatory microglial phenotypes. Conversely, the miRNA miR-124-3p, which is targeted by HOXA11-AS, exhibits an opposing effect, mitigating neuroinflammation and reducing inflammatory damage. Moreover, there is an inverse relationship between the expression levels of Follistatin-like 1 (FSTL1) and miR-134-3p. Additionally, inhibition of the NF-κB pathway has been shown to alleviate neuroinflammation associated with the elevated expression of HOXA11-AS. Based on these findings, it has been hypothesized that silencing HOXA11-AS could potentially reduce the M1 microglial phenotype and suppress neuroinflammatory processes by modulating the miR–124–3p–FSTL1/NF-κB signaling axis (28). The ncRNAs and coding RNA are shown in Figure 2. Overall, the regulatory RNA role is shown in Table 1.

*Small non-coding RNAs (sncRNAs)*

Small non-coding RNAs (sncRNAs) are short, single-stranded RNA molecules that do not encode proteins. Over the past two decades, they have been the focus of extensive research, revealing their crucial involvement in numerous human disorders. sncRNAs participate in a wide array of biological functions, including gene regulation, post-transcriptional modification of mRNA, chromatin remodeling, drug resistance, and the development and progression of cancer. These molecules are classified into various subtypes based on their size and function, such as microRNAs (miRs), small nuclear RNAs (snRNAs), transfer RNAs (tRNAs), and other small RNAs like piwi-interacting RNAs (piRNAs) and small nucleolar RNAs (snoRNAs)(29, 30).

*microRNA*

**Figure 3:**
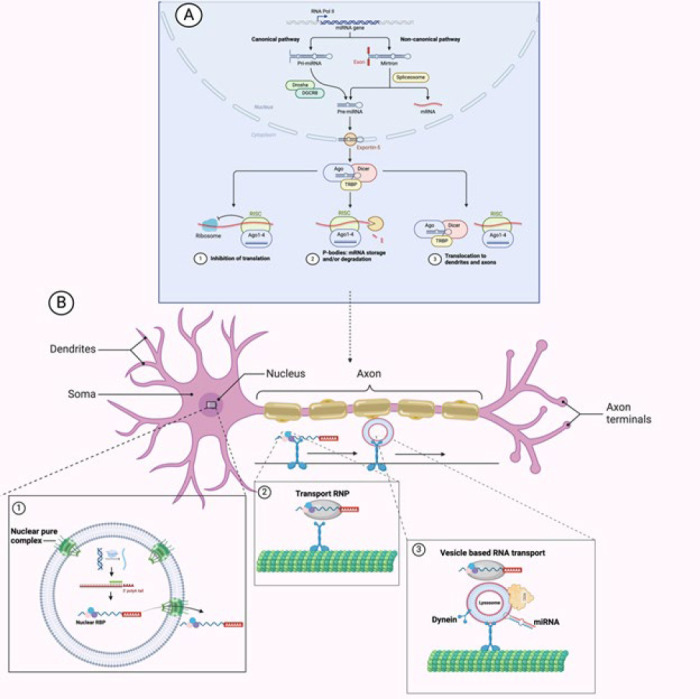
(A) The canonical microRNA (miRNA) pathway involves the generation of primary miRNA (pri-miRNA) transcripts from miRNA genes, which are processed into precursor miRNAs (pre-miRNAs) by Drosha and DGCR8. Alternatively, the mirtron pathway forms intronic pre-miRNA hairpins through splicing and debranching, independent of Drosha. Pre-miRNAs from both pathways are exported to the cytoplasm by Exportin 5, where Dicer processes them into mature miRNAs. These miRNAs are then incorporated into the RNA-induced silencing complex (RISC) with the help of TRBP, leading to the regulation of target mRNAs through translation inhibition or degradation. (B) Ribonucleoprotein (RNP)-mediated transport mechanisms facilitate mRNA movement into axons. RNA-binding proteins (RBPs) form RNPs in the nucleus, which are remodeled and transported through the nuclear pore complex. RNPs associate with kinesin motor proteins or vesicular structures for directed movement along microtubules. Pre-miRNAs and mature miRNAs also utilize vesicular transport systems for delivery into axons, highlighting the importance of RNA mobility in cellular signaling

MicroRNAs (miRs) are small, single-stranded RNA molecules that regulate gene expression by binding to mRNAs’ 3′ untranslated region (UTR), leading to translation inhibition or degradation. Despite their tiny size, their unique solubility and mobility allow for widespread presence in the central nervous system and brain. miRs influence various biological processes, including development, cell differentiation, and metabolism, and they regulate approximately 60% of human protein-coding genes. They are also implicated in cancer, cardiovascular disorders, diabetes, and neurological conditions. miR biogenesis begins with RNA polymerase II transcribing primary-miR (pri-miR) in the nucleus. Drosha and its cofactor DGCR8 cleave pri-miR into precursor-miR (pre-miR), which is transported to the cytoplasm by Exportin-5. There, Dicer further processes pre-miR into a mature double-stranded miR. One strand, the “guide strand,” integrates into the RNA-induced silencing complex (RISC) by binding to Argonaute proteins, while the “passenger strand” is degraded. The guide strand regulates mRNA translation by recognizing complementary sequences in the 3′ UTR, though recent research suggests it may also target the 5′ UTR or open reading frames (ORFs) (31-33). The comprehensive miRNA mechanism in the CNS is shown in Figure 3.

*Piwi-interacting RNAs (piRNAs)*

piRNAs are a category of small non-coding RNAs, roughly 26-31 nucleotides long. They are primarily linked with the PIWI subfamily of Argonaute proteins and play a crucial role in transposon silencing during germline development. Unlike other sncRNAs, piRNAs are generated from a single-stranded precursor without the involvement of the Dicer enzyme. In somatic cells, they form unidirectional clusters, whereas in germline cells, they are organized as dual-stranded clusters. PiRNAs are unevenly distributed across various genomic regions, including repeat sequences, introns, and exons, and are stable in circulation, showing strong expression in body fluids. It is estimated that around 20,000 piRNAs are present in the human genome. They operate through multiple mechanisms, primarily by guiding PIWI proteins to target transposon transcripts, inducing repressive chromatin states at transposon promoters to suppress transcription. Additionally, the PIWI complex can cleave transposon mRNA, leading to the effective silencing of transposons. Recent research indicates that, despite the low levels of piRNAs expressed in somatic tissues, their abnormal expression significantly contributes to cancer development. This suggests that piRNAs could serve as valuable biomarkers and therapeutic targets, as they may possess both tumor-suppressing and oncogenic properties. While much of the specific mechanisms are still not fully understood, considerable research is underway to map their pathways and better define the roles of piRNAs in human diseases (34-37).

*Vault RNA*

Small noncoding vault RNAs (vtRNAs) form a part of the vault complex, a hollow, barrel-shaped ribonucleoprotein complex detected in most eukaryotes (38, 39). Other than the vtRNAs, the vault complex comprises various copies of three protein subspecies: the major vault protein (MVP), the vault poly(ADP-ribose)-polymerase, as well as telomerase-associated protein 1 (40). The various species of organisms exhibit a remarkable diversity in the number of vtRNA paralogs that they express, with the rodentia family demonstrating a rather simplistic genomic configuration by expressing solely a single type of vtRNA, while the more complex human genome is capable of producing multiple variants, namely vtRNA1-1, vtRNA1-2, vtRNA1-3, and vtRNA2-1, thereby indicating a potential evolutionary advantage through this genetic diversification. In addition to this intriguing variation among species, contemporary scientific investigations have strongly stated that vtRNA plays a pivotal role in orchestrating many pathophysiological processes within cellular environments, thus underscoring its significance in cellular biology. In particular, it was revealed through rigorous experimental analysis that the overexpression of vtRNA1-1 not only facilitates the replication of the influenza virus by effectively inhibiting the function of protein kinase R an essential player in the antiviral response but also provides a protective mechanism against cellular apoptosis in models of Epstein-Barr virus (EBV) infection, thereby contributing to the overall understanding of viral pathogenesis. Furthermore, this same vtRNA variant has been shown to influence the intricate process of autophagy, highlighting its multifaceted role in cellular homeostasis and the response to viral challenges, which opens new avenues for research into therapeutic interventions targeting vtRNA functionalities (41).

### Roles for ncRNAs in neurological disorders

**Figure 4:**
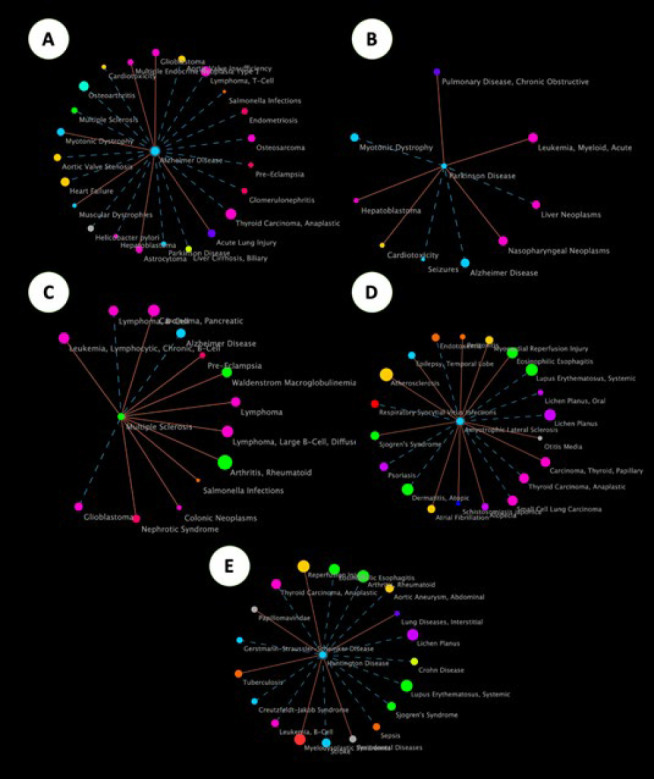
Network maps showing the associations of different diseases with specific molecular or genetic factors across five panels (A-E)

**Figure 5:**
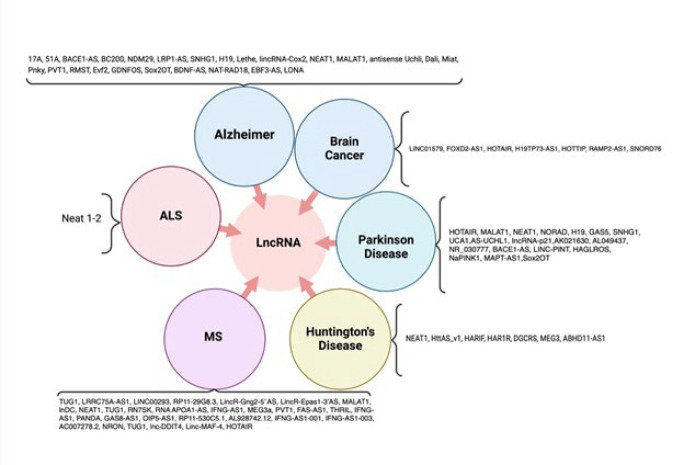
Regulatory role of various lncRNAs against neurodegenerative disorder and brain cancer

ncRNAs play a crucial role in the evolution of brain functions and the central nervous system (CNS) by co-expressing with protein-coding genes, with significant expression in the neocortex and prefrontal regions of the brain. Various molecular mechanisms, including genetic mutations and epigenetic factors, regulate the complexity of neurological disorders, all modulated by ncRNAs. Aberrant functioning of ncRNAs has been identified as a presumptive mechanism in multiple neurological diseases, including Alzheimer’s disease (AD), Parkinson’s disease (PD), Huntington’s disease (HD), and other neuroinflammatory diseases (10, 42, 43). The network map of neurodegenerative disorders is shown in Figure 3. A profound understanding of the role of ncRNAs in the CNS, obtained through elucidating their mechanisms, could lead to innovative therapeutic strategies for treating these diseases (44). In this section, we review the role of ncRNAs, particularly those abundantly expressed in the CNS and significantly altered in AD, PD, and other neuroinflammatory conditions. MiRNAs and lncRNAs are key players in these processes, with miRNAs primarily regulating gene expression at the post-transcriptional level, while lncRNAs interact with miRNAs and other regulatory pathways. Table 2 provides a comprehensive overview of miRNAs in neurodegenerative disorders, and the Network maps of the neurodegenerative disease, as shown in Figures 4 and 5, illustrate the role of various lncRNAs.

*Alzheimer’s disease (AD)*

AD is the most common neurodegenerative disorder that causes dementia. It is characterized by irreversible memory loss and cognitive decline. Patients with AD also experience difficulties with behavioral, language, and executive functions. The disease involves various pathogenic mechanisms, including the deposition of extracellular amyloid β (Aβ) plaques and intracellular neurofibrillary tangles (NFTs). These tangles consist of hyperphosphorylated tau protein, which is a microtubule stabilizer. The presence of Aβ plaques and NFTs is still the primary neuropathologic criterion used for diagnosing AD. In addition, brain atrophy is an inevitable consequence that can occur by accumulating Aβ and NFTs primarily in the hippocampus, neocortex, and other subcortical brain areas. However, the main underlying leading cause of the disease remains undetermined, resulting in no effectual pharmacotherapeutics for the prevention and therapy of AD (45-48). α-secretase-induced cleavage of the amyloid precursor protein (APP), from which Aβ derives, generates peptide p3, whereas β-secretase BACE1 (β-site APP-cleaving enzyme 1)-induced cleavage of the APP produces the soluble APPβ and fragment C99, which eventually γ-secretase takes part in cleaving into a 42-amino acid peptide Aβ42. Mutations in the APP-encoding gene or the presenilin-1 and presenilin-2 genes (components of γ-secretases) can lead to favored cleavage by γ-secretases. These mutations are correlated with the formation of senile plaques and pathological accumulation of Aβ peptide. They also play a significant role in the development of early-onset AD, which occurs before the age of 65 (49, 50).

Synaptic formation and nervous system neuroplasticity are chiefly interrupted by APP, and research has shown several miRs may both directly (miR-16, miR-101, miR-106) or indirectly (miR-9-5p, miR-20a, miR-29a/b-1, miR-124, miR-137, miR-147, miR-181) attenuate its synthesis and also produce of Aβ plaques through targeting the 3′-UTR of APP mRNA. In direct-acting miRs, miR-16 and miR-101 were found to target APP mRNA, inhibiting APP generation; conversely, suppression of miR-101 led to augmented APP expression and Aβ generation. Some of the paracrine effects in the pathogenesis of AD were also shown to be induced by the down-regulation of miR-16 in hippocampal neurons. Also, Optineurin and Sequestosome 1, autophagy receptors, are targeted by miR-9-5p and miR-331-3p, respectively. As autophagy activation could profit the neuronal cell survival, and down-regulated autophagic activity caused by miR-9-5p and miR-331-3p, hence inhibition of which, improves AD. Notably, several studies emphasized the relation of deregulated miR expression (miR-328, miR-15a, miR-15b, miR-195, miR-298, miR-485, miR-103, and miR-107) and raised levels of BACE1, that showed to be increased in AD patients, elevating the risk of the disease (45, 48, 49, 51).

The function of lncRNAs is poorly acknowledged, but it is suggested that they be incorporated into several developmental processes. Lnc 34006 and Lnc-Metastasis Associated Lung Adenocarcinoma Transcript 1 (MALAT1) can result in positive consequences as Lnc34006 is involved in protein ubiquitination pathways and was steeply down-regulated in patients with AD, and also MALAT1 acts as a neuroprotective and anti-inflammatory factor in AD, as confirmed in AD rat models when overexpressed. Some lncRNAs, such as BACE1-antisense transcript (BACE1-AS), BC200, Lnc-NDM29, Lnc-51A, and Lnc-17A, may participate in the pathogenesis of AD. They contribute through various mechanisms. These include enhanced beta-secretase cleavage, mediation of BACE1 activity, and induction of both β- and γ-secretase cleavage. Additionally, they elevate amyloid formation and stimulate inflammatory pathways (47, 52).

*Parkinson’s disease (PD)*

PD is the second most common neurodegenerative disorder after AD. It is characterized by nigrostriatal neurodegeneration due to the intracellular accumulation of Lewy bodies and neurites, primarily composed of α-synuclein (SNCA). Additionally, PD leads to dopaminergic neuron loss in the substantia nigra pars compacta (SNpc). These pathological changes result in progressive and chronic symptoms affecting motor and non-motor functions. Motor symptoms include static tremors, myotonia, bradykinesia, postural instability, and rigidity. Non-motor symptoms include loss of smell, gastrointestinal issues such as constipation, depression, dementia, anxiety, sleep disorders, and dysautonomia. Despite the enormous accomplishments in the understanding of PD that have been done in the more than 200 years of PD research and evidence indicating the close relationship between the development of PD and disruption in apoptosis, oxidative stress, autophagy, cell survival, protein aggregation, mitochondrial function, and neuroinflammation, the underlying etiology and mechanism remains to be elucidated (53-55). 

Neuronal immune system activation involves microglia, inflammatory cytokines, and astrocytes. This process results from cellular stress (e.g., oxidative stress) and environmental factors (e.g., diet or age). These factors trigger a persistent cycle of neuroinflammation, creating a chronic inflammatory environment that endangers neurons. Ultimately, this can lead to neuronal apoptosis, a well-established PD mechanism. Additionally, several genes were reported to be dysregulated in the disease, such as SCNA, DJ-1, leucine-rich repeat kinase 2 (LRRK2), PTEN-induced putative kinase 1 (PINK1), and PARKIN, causing a familial form of PD. Nuclear receptor-related 1 (Nurr1) and GBA are the rare genes to be mutated in less than 5% of patients with PD (56, 57).

Neurodegenerative processes, apoptosis, and regeneration are the outcomes of the pathways targeted via miR-195, miR-24, and miR-19b, which could also be utilized as a diagnostic tool. Also, disorders of the Let-7 miR family, consisting of let-7a to let-7k, miR-98, and miR-202, can result in many complications, including neurodegenerative diseases, notably overexpressed in PD models, acting through activation of toll-like receptor 7 (TLR7). Nevertheless, in the plasma of the patients who have not undergone any therapy, let-7a and let-7f were found to be down-regulated. miR-133 was one of the first miRs to be explored, as it showed reduced levels in the midbrain of PD patients, which acts as a regulatory factor in the maturation and function of dopaminergic neurons through a negative feedback circuit, which contains the paired-like homeodomain transcription factor Pitx3. Furthermore, studies report identifying six more miRs to be disrupted in the setting of PD, namely miR-1, miR-22, and miR-29a (reduced levels in treatment-naive patients in comparison with healthy controls), and miR-16-2, miR-26a-2, and miR-30a (enhanced levels in treated patients in comparison with treatment-naive patients) (58-61).

lncRNAs are demonstrated to be regulated in PD as HOX transcript antisense RNA (HOTAIR) up-regulates LRRK2 in PD development, promoting the PD phenotype induced by 1-methyl-4-phenyl-1,2,3,6-tetrahydropyridine (MPTP) in mice and 1-methyl-4-phenylpyridinium (MPP) in SH-SY5Y cell lines, representing a protective effect against neuroinflammation. Additionally, MALAT1 is an emerging potential target involved in apoptosis regulation. It up-regulates α-synuclein protein expression in an MPTP-induced PD mouse model and SH-SY5Y cells treated with MPP+. MALAT1 interacts with the enhancer of zeste homologue 2 (EZH2) to regulate the nuclear factor (erythroid-derived 2)-like-2 (Nrf2)-mediated antioxidative response. This process ultimately activates inflammasome microglial cells (62, 63). Moreover, up-regulated levels of small nucleolar RNA host gene 14 (SNHG14) were reported in brain tissues of PD mice, which also decreased dopaminergic neuron injury resulting from silencing SNHG14 expression, which acted by targeting miR-133b, a factor closely associated with PD progression (64).

*Multiple sclerosis (MS)*

The high expression of miR-326 in Th17 cells derived from the peripheral blood of patients with relapsing-remitting multiple sclerosis (RRMS) was associated with increased IL-17 levels and greater disease severity (65). The overexpression of miR-326 increases the prevalence of Th17 cells and induces significant experimental autoimmune encephalomyelitis (EAE) by down-regulating Ets-1, which acts as a negative regulator of Th17 cell development. In contrast, knocking down miR-326 reduces EAE severity and suppresses Th17 levels. Additionally, miR-155 in T-lymphocytes promotes Th17 cell development and function by inhibiting Ets-1 transcription (66, 67). In relapsing MS cases, miR-590 expression is elevated, promoting Th17 cell differentiation by targeting Tob1 (Transducer of Erb-2), a protein from the Tob/BTG family known for its anti-proliferative properties (68). Moreover, miR-590 influences the pathogenicity of Th17 cells by enhancing the expression of inflammation-related molecules such as CXCL3, CSF2, and IL-23R. In addition, miR-448 may facilitate Th17 cell development by directly suppressing the anti-inflammatory enzyme protein tyrosine phosphatase non-receptor type 2 (PTPN2) (69). In both experimental EAE and MS cases, the let-7e miRNA showed a marked increase in expression. Inhibiting let-7e drives the immune response toward a Th2 profile, leading to a reduction in MS severity. Conversely, elevated let-7e levels enhance a Th1/Th17 response, exacerbating EAE symptoms (70). Studies have demonstrated that reduced levels of specific miRNAs impact the differentiation of Th1 and Th17 cells. For example, miR-15b targets O-linked N-acetylglucosamine transferase, a crucial enzyme for activating CD4+ T cells and triggering hyperinflammatory responses (71). In MS patients, miR-15b expression is lowered to inhibit the development of Th1 and Th17 cells. Elevating miR-15b levels has been shown to relieve symptoms in experimental EAE, whereas sustained suppression of miR-15b aggravated the condition. Likewise, the miR-132 cluster is expressed at lower levels in CD4+ T cells (72). Bcl-6 is a target of miR-140-5p and acts as a suppressor of Th17 differentiation, with its reduction influenced by EAE severity. Typically, miR-140-5p inhibits Th1 cell development by down-regulating the signal transducer and activator of transcription 1 (STAT1). However, in MS, miR-140-5p expression is notably reduced, promoting Th1 cell growth and worsening MS severity (73).

In MS, abnormal miRNA expression is observed in Th cells and various other T cell subsets, affecting their polarization. For instance, miR-128 and miR-27b are up-regulated in naïve CD4+ T cells, whereas miR-340 shows heightened expression in CD4+ memory T lymphocytes (74). These miRNAs impede Th2 cell differentiation by directly down-regulating IL-4 and BMI1 (B lymphoma Mo-MLV insertion region 1 homolog), thus promoting a shift toward Th1 responses. Notably, applying oligonucleotides targeting these miRNAs can re-establish Th2 responses in individuals with MS (75). In CD4^+^ memory T cells, miR-29b, which is triggered by IFN-γ, functions in a negative feedback loop through suppressing T-bet and IFN-γ transcription to manage Th1 cell bias (76). In MS, Treg cells show varied expression in 23 miRs compared to healthy controls (77). Among the remarkably enhanced miRs was miR-106b/25, which mediated the TGF-β pathway essential for developing Th17/Treg cells (78). TGF-β signaling is also enhanced in pro-inflammatory CD8^+^ T-lymphocytes by promoting miR-629 (79). Together, these findings suggest that miRNAs play a role in modulating the differentiation of proinflammatory Th1/Th17 cells, developing regulatory T cells (Tregs), and balancing Th2 and Th1 responses in MS by targeting specific genes for suppression. Beyond T cells, several crucial miRNAs are also produced differently in MS B-cells, with miR-320a showing a notably reduced expression in B-lymphocytes (80). Among its various targets, miR-320a inhibits matrix metallopeptidase-9 (MMP-9) produced by activated B cells. In MS, the reduction of miR-320a leads to increased expression and secretion of MMP-9 in B cells, disrupting the blood-brain barrier (BBB) and damaging myelin basic protein (MBP)(81). Similarly, the significantly increased expression of miR-132 in MS cases reduces Sirtuin-1 levels in B cells, which contributes to the elevated production of pro-inflammatory cytokines such as lymphotoxin and tumor necrosis factor (TNF-α)(82). miR-17–92 inhibits the expression of Bim, a pro-apoptotic protein, and PTEN, a tumor suppressor. Consequently, a decrease in miR-17–92 results in increased Bim levels, which hinders pro-B cell maturation into pre-B cells (83). In untreated RRMS patients, both circulating CD14+ monocytes and CD68+ cells from active lesions, including perivascular (blood-derived macrophages) and parenchymal (microglia) cells, exhibited significantly higher levels of miR-155 compared to controls. Consequently, the increased expression of miR-155 in these cells was associated with elevated secretion of pro-inflammatory cytokines (84). miR-124 is considered a key regulator of microglial quiescence. In EAE, activated microglia show reduced levels of miR-124. Conversely, increasing miR-124 expression may induce a state in activated microglia that resembles quiescence, helping to prevent EAE by decreasing macrophage activity through the C/EBP-α-PU.1 signaling pathway (85). Overall, these studies demonstrate the crucial impact of miRs on processes modulated by antigen-presenting cells (APCs) by influencing their activation and effector functions. Similar to immune cells, the abnormal expression of miRs in the nervous system contributes to the hyperinflammatory processes associated with MS. In a study analyzing cell-type-specific miR profiles, 10 miRs were significantly elevated in active MS lesions (86). CD47, which is widely expressed in various human cells to prevent phagocytosis, is reduced in the active lesions of MS patients. Three miRs, such as miR-155, miR-34a, and miR-326, are elevated in MS and may target CD47, thereby releasing macrophages from inhibitory control and promoting myelin degradation. These changes primarily occur in astrocytes. Furthermore, miR-155 directly targets and reduces SOCS1, a negative regulator of cytokine production in astrocytes (87). miR-155 influences the neurosteroid synthesis enzymes ARK1C1 and ARK1C2 (88). A previous study identified that miR-23’s modulation of lamin B1 is crucial for the growth and myelination of oligodendrocytes, suggesting its potential involvement in the pathogenesis of MS. In MS patients, miR-219 and miR-338 levels are decreased compared to controls (89). Elevated levels of miR-219 and miR-338 enhance the differentiation of oligodendroglial precursor cells (OPCs) in culture. Recent research involved introducing these miRs into the oligodendrocytes of rodents, demonstrating their potential to promote oligodendrocyte differentiation and myelination. Their findings suggest that the dysregulation of miRs creates an environment that supports remyelination and axon regeneration, both of which are compromised in MS (90).

The investigation into the altered expression of lncRNAs has been rigorously conducted within various biological fluids, including serum, plasma, peripheral blood mononuclear cells (PBMCs), and whole blood, particularly focusing on patients diagnosed with relapsing-remitting multiple sclerosis (RRMS) and secondary progressive multiple sclerosis (SPMS) as indicated in reference 83. In a manner analogous to miRs, lncRNAs possess the capacity to modulate and influence the functional dynamics of a diverse array of immune cell types within the immune system. Specifically, in individuals who have multiple sclerosis, the heightened expression levels of NEAT1 have been observed to instigate the reorganization of the splicing factor known as proline/glutamine-rich (SPFQ) from the promoter region of IL-8, which subsequently leads to the transcriptional activation of the IL-8 gene, as discussed in reference 84. Furthermore, the lncRNA known as RN7SK plays a pivotal role in the regulatory processes governing CD4+ T lymphocytes and is implicated in the exacerbation of hyperinflammatory responses, as noted in reference 85. The increased expression of RN7SK RNA within the context of the 7SK small nuclear ribonucleoprotein (snRNP) complex can inhibit the action of P-TEFb catalytically. This kinase complex comprises Cdk9 and cyclin T1, both of which are crucially involved in the differentiation processes of CD4+ T cells. Additionally, the lncRNA TUG1 has been found to exhibit elevated levels in a variety of neurodegenerative conditions, with multiple sclerosis being among them. Notably, the promoter region of TUG1 harbors conserved binding sites for the p53 tumor suppressor protein and functions as a downstream target of p53, thereby playing a significant role in the signaling pathways associated with apoptosis. In summary, the intricate interplay between these lncRNAs and various cellular processes underscores their potential significance in the pathophysiology of multiple sclerosis and other neurodegenerative diseases. The elucidation of these molecular mechanisms may provide invaluable insights into novel therapeutic strategies aimed at modulating immune responses and cellular functions in affected patients.

Altered lncRNA expression has been investigated in serum, plasma, PBMCs, and blood, specifically in patients with RRMS and secondary progressive (SP) MS (91). Similar to miRs, lncRNAs can influence the functions of various immune cells. In MS patients, elevated levels of NEAT1 cause the rearrangement of the splicing factor proline/glutamine-rich (SPFQ) from the IL-8 promoter, resulting in the transcriptional activation of IL-8 (92). *RN7SK *regulates CD4+ T lymphocytes and contributes to hyperinflammation (93). The up-regulation of RN7SK RNA in the 7SK snRNP complex may catalytically inhibit P-TEFb, a kinase consisting of Cdk9 and cyclin T1, which is involved in differentiating CD4+ cells. TUG1 is elevated in various neurodegenerative disorders, including MS. The promoter of TUG1 contains conserved p53-binding sites and is a downstream target of p53, playing a role in apoptotic signaling (94). Additionally, the lncRNA growth arrest-specific 5 (GAS5) is up-regulated in amoeboid-shaped microglia in MS patients (95). GAS5 has been demonstrated to facilitate the polarization of microglia towards the M1 phenotype, resulting in demyelination. The ablation of GAS5 in transplanted microglia was associated with a deceleration of MS progression *in vivo* and enhanced remyelination. GAS5 exerts an inhibitory effect on T-lymphocyte proliferation by interacting with PRC2, while concurrently suppressing the activity of the transcription factor IRF4. Notably, there was a significant increase in levels of linc-MAF-4 observed in peripheral PBMCs derived from patients with MS. Linc-MAF-4 plays a pivotal role in the pathogenesis of MS by modulating the Th1/Th2 balance and targeting MAF, a transcription factor essential for the differentiation of Th2 cells (96). Given their ability to activate or inhibit gene expression through various pathways, it is clear that lncRNAs serve as crucial mediators in the pathogenesis of MS. They are also strong candidates for biomarkers in MS detection due to their stability in body fluids and specificity to specific cell types. In 2024, researchers found that miRs are dysregulated in PBMCs from MS patients and are linked to T-cell regulators. The T-lymphocyte mediator FOXP3 was elevated in the PBMCs of MS patients, and out of the 21 miRs investigated in that study, 13 were dysregulated in MS cases, largely regardless of MRI disease activity or treatment. Several miRs showed positive or negative associations with IL21 and FOXP3. Additionally, serum levels of the neurofilament light chain were increased in active MS cases but did not correlate with any specific miR or mRNA (97). miR-33-3p, miR-34c-5p, and miR-124-5p inhibit the differentiation of oligodendrocyte progenitor cells (OPCs) during the late progenitor stage, whereas miR-145-5p acts at the pre-myelination phase to suppress differentiation. In contrast, miR-214-3p promotes the differentiation of CG-4 cells (98). 

A recent study found that the rs2431697 polymorphism in the miR-146a gene was not associated with MS. However, a significant relationship was observed between the rs2910164 polymorphism of the miR-146a gene and MS in the examined sample (99). In 2024, another study revealed a significant positive correlation between miR-155 (rs767649 A>T) genotypes, miR-155 expression, and susceptibility to MS in Iraqi patients (100).

The previously uncharacterized ncRNAs, specifically vtRNA 1-1 and vtRNA 2-1, were examined in a cohort of 32 blood samples. Investigators identified notable disparities in the expression profiles of vtRNAs between blood samples obtained from patients with PPMS/RRMS and those from healthy controls. Should these observations be substantiated through analysis of a larger population, vtRNAs may be recognized as potential diagnostic indicators for multiple sclerosis (101). 

*Huntington’s disease (HD)*

HD is a fatal, progressive, incurable, and hereditary neurodegenerative disease, identified by atypical extended CAG repeats in the huntingtin (HTT) protein-encoding gene, resulting in a gain of function mechanism and death of the neurons, subsequently leading to the hallmark of the disease, which is the mutant HTT N-terminal fragments neuronal inclusions, primarily in the striatum and cortex (102, 103). Neurodegeneration in patients occurs many years before the onset of diagnostic symptoms and signs of HD appear, which include cognitive and motor changes, such as memory loss, mental deterioration, emotional problems, sleep disorders, involuntary movements, difficulty speaking, walking and swallowing, grimace, and also chorea, which is the most distinct motoric symptom (104-106).

Altered neuronal and non-neuronal cell gene expressions play a significant part in HD pathogenesis. Epigenomic and transcriptional modulators such as Repressor element 1-silencing transcription factor (REST), RE1 (Repressor element-1), CBP (CREB-binding Protein), TBP (TATA-Box Binding Protein), and P53 were reported to interact with HTT, as a cytoplasmic complex of HTT-associated protein 1 (HAP1) aids HTT in blocking REST-mediated transcriptional inhibition, leading to the assembly of the repressor complex in the nucleus in mutated HTT (mHTT). Moreover, a reduction in the expression of Brain-Derived Neurotrophic Factor (BDNF) has been noted; this neurotransmitter regulator plays a crucial role in neuroplasticity and is pivotal for growth and survival. Methylation of histone 3 lysine 27 (H3K27) is catalyzed by polycomb repressive complex 2 (PRC2), functioning as methyltransferase associated with chromatin, which acts as one of the primary targets of the mutated genes (102, 107).

Preponderating mRNA deregulations, principally in the cortex and striatum, are reported to be a contributing factor in HD pathogenesis, and changes in post-transcriptional miR-mediated regulation are a major mechanism resulting in mRNA dysregulation in HD (43). miR-9-5P modulates neuronal fate, inhibiting REST expression in a negative feedback loop as REST suppresses neural fate and stimulates the expression of miR-9-5p. Interestingly, the aberration of this mechanism is noted in HD pathogenesis (108). miR-22 is a prominent modulator in the neuronal survival pathway and neurogenesis in HD, targeting REST co-repressor 1 (Rcor1), histone deacetylase 4 (HDAC4), and regulator of G-protein signaling 2 (Rgs2), all of which can act as protective modulators in HD pathogenesis. Moreover, the expression of pro-apoptotic proteins, namely tumor protein p53-inducible nuclear protein 1 (Tp53inp1), and mitogen-activated protein kinase 14/p38 (MAPK14/p38) has been reported to be directly inhibited via miR-22. Additionally, via an unknown mechanism, miR-22 can reduce the aggregation of mHTT. However, studies have shown miR-22 decreases in the disease setting (109). miR-124 and miR-132, targeted by REST, were among the miRs that were reported to decrease in HD patients. Therapeutic effects were shown in the setting of miR-124 overexpression in transgenic HD mice via RE1-silencing transcription factor. In addition, miR-132 performs by inhibiting a neurite growth factor, targeting rho GTPase-activating protein 32 (110, 111).

lncRNAs act as vital modulators in HD, interacting with several factors. Nuclear paraspeckle assembly transcript 1 (NEAT1), Taurine up-regulated gene 1 (TUG1) could be mentioned as up-regulated lncRNAs in HD, which play their roles as protectors of neurons against damage via interacting with p53 and ubiquitin-proteasome system, and also promoting neuron survival against mHTT through chromatin remodeling by binding to PRC2 and p53, respectively. Furthermore, maternally expressed gene 3 (MEG3), Human accelerated region 1 (HAR1), and HTT antisense RNA (HTT-AS), noted to be down-regulated in patients with HD. MEG3 down-regulation may be correlated to the pathological process of mHTT, in which its expression is inhibited due to being targeted by REST, as reported in the HAR1 mechanism. Also, HTTS-AS acts by directly inhibiting the HTT expression (112, 113).

*Amyotrophic lateral sclerosis (ALS)*

ALS is a progressive neurodegenerative disorder described by the death of the predominantly motor neuron cells in the CNS, resulting in atrophy and weakness of voluntary skeletal muscles, and the lethality of the disease commonly occurs 3-5 years following diagnosis. ALS can affect either upper motor neurons (UMN) or lower motor neurons (LMN). However, both UMN and LMN are affected in 70% of cases, presenting with the symptoms of limb weakness or bulbar manifestations, such as difficulty in swallowing or speech (114, 115). 

Familial ALS (FALS) consists 5-10% of all ALS cases, of which 25-40% and a slight portion of sporadic ALS developed by several processes taking part in ALS, including an intronic expansion of hexanucleotide repeat in the gene coding chromosome 9 open reading frame 72 (C9ORF72), and also mutations in superoxide dismutase 1 (SOD1)*, *TAR DNA binding protein 43 (TDP-43), and Fused in Sarcoma (FUS). Furthermore, in addition to affected neuronal cells, microglia have* also* been shown to possess SOD1 mutations, leading to the degeneration of motor neurons, and the vast majority of both sporadic and familial cases have been shown to exhibit aggregations of neuronal TDP-53 protein (116, 117).

Neuronal activity, survival, and differentiation have been demonstrated to be driven by several miRs, such as miR-206, miR-155, miR-142-5p, miR-27a, miR-34a, miR-4299, and miR-4649-5p, whose recognition has been a priority for the evolution of proper therapy. The lifespan of the mouse model was noted to be remarkably prolonged due to the decrease in the miR-155 expression, an inflammation-associated factor, which was also up-regulated in the mouse with the mutant SOD1 gene (43, 118, 119). Another implying factor is miR-206, a skeletal muscle-specific miR that acts through the induction of the secretion of fibroblast growth factor binding protein 1 (FGFBP1) from muscles by hindering the translation of HDAC4, resulting in the promotion of synaptogenesis and ultimately being a beneficial factor for healing ALS, as the disease aggravated with a miR-206 knockdown in SOD1 mice. miR-4299 and miR-4649-5p are potential ALS biomarkers for diagnosis, as their down-regulation and up-regulation were unaffected by clinical characteristics. In addition, miR-124 has been shown to be a vital factor in neuron and astrocyte differentiation in ALS transgenic mice by targeting Sox2 and Sox9, which control the neural stem cell differentiation into astrocytes throughout the disease pathogenesis (109, 119, 120).

Various lncRNAs have been reported to have a role in ALS pathogenesis, namely NEAT1, MALAT1, MEG3, and C9ORF72 antisense transcript (C9ORF72-AS), which regulate through targeting mRNAs. For instance, MEG3 targets Hox, resulting in the PRC2-Jarid2 complex formation. (G4C2)n repetition in C9ORF72 has been recognized as one of ALS’s most frequent genetic factors. Noted repetitions in C9ORF72-AS can form a C-rich sequence, influencing the transcription and stability of the genome. Additionally, NEAT1_1 and NEAT1_2 are the two lncRNAs expressed via the NEAT1 promoter, which can be utilized as a biomarker for the disease, and also in therapeutic strategies such as preserving an appropriate NEAT1_2 expression for the formation and maintenance of a specific nuclear structure named paraspeckles. One of the most efficacious treatments for ALS can be deleting free NEAT1_1 as it regulates driving neurons into apoptosis. Also, ameliorating the toxicity of TDP-43 is the mechanism through which MALAT1 functions (113, 121).

### Roles of ncRNAs in brain cancers

**Figure 6:**
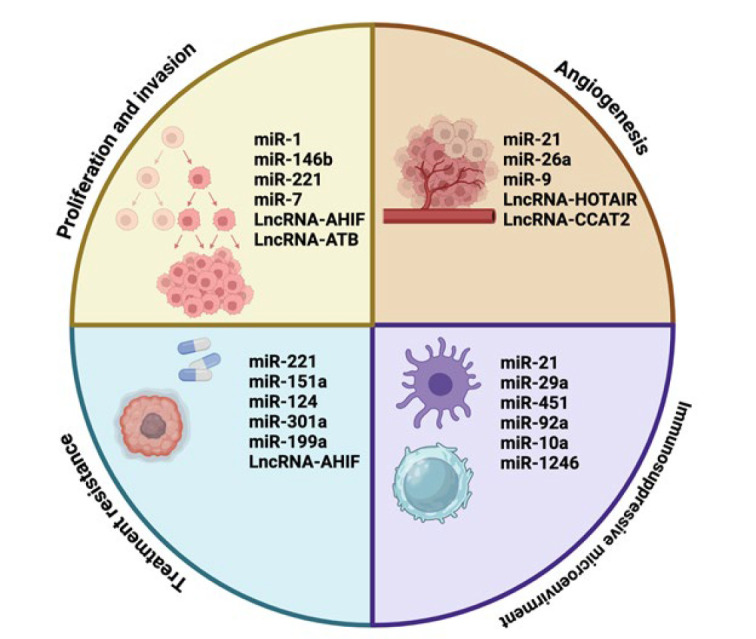
Role of exosomal non-coding RNAs in glioma biology

ncRNAs, particularly miRs and lncRNAs, play a crucial role in the development and progression of various cancers, including glioma, a complex brain cancer with limited therapeutic options (122). Research findings undeniably demonstrate that ncRNAs are disrupted in the context of brain cancer, consequently exerting an influence on a broad spectrum of cellular mechanisms pivotal to both metastasis and growth (123). This influence primarily stems from their capability to modulate gene expression through interactions with mRNAs and proteins, thus impacting vital signaling pathways implicated in glioma progression (124). Moreover, ncRNAs, apart from their regulatory functions related to apoptosis in brain tumors, have been subject to comprehensive investigation as prospective indicators for the diagnosis and prognosis of gliomas. The dysregulated expression of specific lncRNAs and miRs has been found to correlate with glioma grade, prognostic outcomes, and responses to treatment. In summation, ncRNAs have solidified their position as integral contributors to the intricate landscape of glioma development and progression (125, 126). The important role of the exosomal ncRNAs is demonstrated in Figure 6. Traditional histopathological examination of biopsy samples faces inherent limitations due to the complexities associated with surgical procedures. Moreover, this approach may fail to fully encompass the extensive genetic heterogeneity of GBM cells, rendering it suboptimal for a comprehensive molecular characterization of the tumor (127). Exosomes, by contrast, possess a unique capability to traverse the BBB and other anatomical compartments through transcytosis, thereby gaining access to the circulatory system and facilitating their systemic distribution (128). Exosomes enriched with specific ncRNAs exhibit remarkable stability, high abundance, reproducibility, and disease specificity. These characteristics enable them to circumvent the constraints associated with tissue-specific miRNAs, which are detectable only within the tumor microenvironment. Consequently, exosome-derived ncRNAs serve as a promising foundation for developing liquid biopsy strategies in GBM (129).

miRNAs have emerged as highly promising candidates for glioma diagnostic and prognostic biomarker development. In a study investigating glioma patients undergoing radiotherapy, Li *et al*. employed miRNA sequencing to analyze serum-derived exosomal miRNAs. Their analysis revealed a distinct profile of differentially expressed (DE) miRNAs, comprising 18 up-regulated and 16 down-regulated species, highlighting their potential role in glioma pathophysiology and treatment response assessment (130). Interestingly, miR-454-3p exhibited a contrasting expression pattern, showing significant down-regulation in glioma tissues while being markedly up-regulated in serum-derived exosomes from the same patients (131), suggesting its potential as a non-invasive biomarker for monitoring glioma progression. MiR-21-5p expression was higher in GBM tissues than in lower-grade gliomas and normal brain tissue, while miR-9-5p and miR-124-3p were overexpressed in exosomes derived from GBM stem cell lines (132). Elevated expression of miR-454-3p in exosomes, or its reduced levels in tumor tissues, was correlated with a poorer prognosis. Likewise, intratumoral miR-181d expression showed a positive association with favorable functional parameters in glioma patients, whereas higher levels of exosomal miR-181b were linked to deteriorated functional outcomes (133). Remarkably, increased levels of exosomal miR-181b were strongly linked to a significant reduction in postoperative survival and an exacerbation of tumor-associated symptoms. Similarly, serum miR-301a expression was found to be elevated in glioma patients, with its levels progressively rising in correlation with tumor grade. Following the primary tumor resection, serum exosomal miR-301a levels markedly declined but surged again upon glioblastoma recurrence, reinforcing its potential as a dynamic biomarker for tracking glioma progression.

These shifts in exosomal miRNA expression patterns underscore their potential as glioma progression and therapeutic response indicators. For example, Chun *et al*. reported fluctuations in human chorionic gonadotropin (hCG) and annexin A5 levels under temperature-induced stress, suggesting their viability as biomarkers for monitoring GBM cell responses to external environmental factors (134). Wang *et al*. identified 109 up-regulated and 61 down-regulated miRNAs in serum exosomes from patients with intracranial lymphoma and high-grade glioma (135). Among these, miR-766-5p and miR-376b-5p emerged as promising biomarkers for distinguishing high-grade glioma from intracranial lymphoma. Furthermore, Santangelo *et al*. reported that serum exosomal levels of miR-21, miR-222, and miR-124-3p were significantly elevated in patients with high-grade glioma compared to those with low-grade gliomas or healthy individuals. Notably, these levels declined sharply following surgical resection, highlighting their potential utility in both glioma diagnosis and post-surgical monitoring (136). Moreover, the lncRNA HOTAIR exhibited significant up-regulation in serum samples from GBM patients. Its heightened expression was strongly associated with high-grade brain tumors, indicating its potential as a novel biomarker for both the diagnosis and prognostic assessment of GBM (137).

In addition to miRNAs, proteins, and mRNAs involved in immune responses, plasma exosomes are emerging as valuable biomarkers for evaluating glioma patients’ responses to immunotherapy. A study by Muller *et al*. revealed that higher levels of exosomal IL-8 and TGF-β mRNA were associated with enhanced immunologic responses following vaccination in glioma patients. In contrast, PD-1 mRNA levels remained consistently elevated, highlighting the potential of these markers for tracking the effectiveness of immunotherapy (138). Survivin (SVN), an inhibitor of apoptosis protein, plays a role in cancer cell proliferation, immune suppression, and chemotherapy resistance, positioning it as a promising cancer biomarker (139). A study involving malignant glioma patients treated with the anti-SVN vaccine found that exosomes marked with CD9+/GFAP+/SVN+ and CD9+/SVN+ were present in the bloodstream. Notably, a decrease in these exosomes early on was linked to extended progression-free survival, underscoring their potential as indicators for monitoring treatment response (139). 

*miRs*

The operational mechanisms of miRs in the context of brain cancer are characterized by their multifaceted involvement with a range of signaling pathways, of which the transforming growth factor-beta (TGF-β)/bone morphogenic protein (BMP) signaling cascade holds significance, particularly in the context of bone development and osteoblast lineage. MiRs possess the capacity to regulate this pathway, thereby impacting the differentiation and functions of osteoblasts, which is relevant for brain cancer cells that display characteristics of osteoblastic differentiation (141). While glioblastoma is primarily considered a tumor of neural origin, emerging evidence suggests that glioblastoma stem cells (GSCs), the cancer-initiating cells responsible for tumor growth and recurrence, exhibit remarkable plasticity and the ability to differentiate along mesenchymal lineages, including into osteoblast-like cells under specific conditions (142). This unexpected differentiation potential has been observed in experimental studies, where exposure to specific environmental cues, such as bone morphogenetic proteins (BMPs), has induced GSCs to adopt osteogenic characteristics (143). This phenomenon has potential therapeutic implications. Since GSCs drive glioblastoma progression and treatment resistance, forcing their differentiation into osteoblast-like cells or other non-proliferative cell types may serve as a strategy to reduce their tumorigenic capacity. Several studies have explored the use of differentiation therapy, wherein GSCs are induced to exit their stem-like state and transition into lineages with lower malignant potential. While this approach remains experimental, it aligns with broader efforts in cancer research to exploit cellular plasticity as a therapeutic target (144). However, the molecular mechanisms governing this differentiation remain incompletely understood. Research suggests that BMP signaling pathways and other regulatory factors may play key roles in driving mesenchymal transitions in GSCs. Further investigation is needed to elucidate the precise signaling mechanisms and assess whether differentiation-based therapies could be a viable strategy for glioblastoma treatment (144).  Furthermore, miRs can engage with diverse cellular constituents, including mesenchymal stromal cells (MSCs), extracellular vesicles, and factors linked to inflammatory processes. Through these interactions, they exert control over the microenvironment within the tumor, thereby exerting an impact on the advancement of the tumor (146). Over the past decade, extensive research has uncovered numerous miRs with both oncogenic and tumor-suppressive attributes in the context of malignant glioma. Among these, miR-21 stands out as the primary and most broadly researched prospect, presenting considerable up-regulation in glioma compared to normal brain tissues. In contrast, suppressed miR-21 has resulted in a reduction in invasiveness, cell proliferation, tumorigenic potential, and an upsurge in apoptosis by involving key components such as PDCD4, MMP, TIMP3, RECK, and insulin-like growth factor-binding protein-3 (IGFBP3)(145).

Regarding other notable miRs in glioma, miR-218 showed diminished expression levels compared to normal tissues by modifying the IKKβ’s 3’UTR length, leading to alteration of NFκB’s transcriptional activity (146). Also, the MIR31HG gene, located in close proximity to CDKN2A/B on chromosome 9, encodes miR-31, which has been dysregulated in over 72% of GBM cases, and also reduces the tumor’s load by suppressing NF-κB signaling, regardless of the status of CDKN2A/B, when introduced (147). Also, in other types of brain neoplasms, such as pituitary tumor and prolactinoma, studies suggest contradictory results as miRs, including miR-145 and miR-34, showed heightened responsiveness of prolactinoma cells to bromocriptine by amplifying the expression of TPT1, and up-regulation, leading to proliferation suppression of pituitary tumor cells, promoting cell apoptosis by modification of SOX7 expression, respectively. On the other hand, another study found elevated levels of miR-145 and miR-34a in AIP-mutation-positive somatotropinomas in comparison with AIP-mutation-negative ones, highlighting a crucial connection between the signature of miRs and AIP-mutation (148, 149).

*lncRNAs*

LncRNAs have been observed to have a substantial impact on a range of cancer types, including brain cancer (18). Specific lncRNAs have been pinpointed in brain cancer, deeply entwined with pivotal roles in steering the growth and advancement of brain tumors, exhibiting dual capabilities, functioning as either oncogenes or tumor suppressors (151, 152). Notably, Colorectal Neoplasia Differentially Expressed (CRNDE), the lncRNA with the most pronounced up-regulation in gliomas, has been the subject of extensive mechanistic investigations unveiling the up-regulation of CRNDE via the potential influence of histone acetylation within the promoter region, leading to oncogenic activities of glioma stem cells (153), in which the negative regulation of miRs, such as miR-186 yielded some oncogenic effects of CRNDE (154). Moreover, *in vivo* studies unveiled the intricate CRNDE/miR-29c-3p interactions in medulloblastoma, which led to negative regulation of miR-29c-3p, subsequently influencing apoptosis, invasion, migration, proliferation, and the resistance of tumors to chemotherapeutic agents (155). Additionally, in a separate investigation exploring H19’s function in human glioblastoma, it was demonstrated that overexpression of H19 drives glioblastoma invasion, stemness, tumorigenicity, and angiogenesis, eventually aligning with patient survival outcomes (156). Also, research shows that HOTAIR, which is a critical regulator of cell cycle progression, associates along with several signaling pathways, such as the Wnt/β-catenin axis, and also interacts with related miRs, like miR-126-5p, miR-15b, miR-141, miR-148b-3p, and miR-326, highlighting its pivotal oncogenic role in glioblastoma (157). HOTAIR overexpression was observed to be significantly correlated with the glioblastoma grade, as the serum of the patients contained markedly higher levels than the control group (158). Nevertheless, it was revealed that in pediatric ependymomas, lower levels of HOTAIR were expressed in the serum of the patients (159). Moreover, studies investigating the role of X-Inactive Specific Transcript (XIST), one of the earliest discovered lncRNAs, demonstrated the correlation between miR-137 and XIST, in which XIST down-regulates miR-137 leading to up-regulation of Rac1, resulting in glioma cell proliferation (160). Also, XIST enhanced the expression of cyclic AMP response element-binding protein 1 (CREB1) through sequestering miR-329-3p, leading to apoptosis suppression and reducing the sensitivity of glioma cells to radiation (161), contributing to unfavorable clinicopathologic characteristics and reduced survival duration (162). 

Studies have also focused on the deregulations of maternally expressed gene 3 (MEG3), a lncRNA situated within the imprinted delta-like homolog 1 gene‒type III iodothyronine deiodinase gene (DLK-DIO3) region, revealing a decrease in MEG3 expression in glioma cells, diminishing its tumor-suppressive role, resulting in heightened proliferation, invasion, and migration, and ultimately resulting in unfavorable patient survival outcomes (163, 164). Mechanistically, these effects involve the attenuation of Wnt/β-catenin signaling and direct binding to miR-19a, which suppresses tumorigenesis by acting as a competitive endogenous RNA (165, 166), potentially influencing RB1, GDF15, TP53, MDM2, and some other key factors (167). Additionally, a separate study revealed the negative correlation between the glioma grade and the level of MEG3, establishing it as an independent prognostic indicator (164).

Moreover, research studying the role of taurine up-regulated gene 1 (TUG1) indicated the overexpression of which in glioma cells, thoroughly through binding to miR-144 (168), inhibiting miR-26a (169), and miR-299 (170), leading to increased blood-tumor barrier permeability which restricts the transportation of chemotherapy drugs to brain tumor tissues, up-regulation of phosphatase and tensin homolog (PTEN), and boosting the tumor-driven angiogenesis by VEGF expression, respectively, highlighting TUG1 as a promising therapeutic target for the treatment of glioblastoma (170). In a separate investigation, it was intriguing to note that TUG1 exhibited decreased levels in glioma, which was associated with GATA6-AS, a regulator of endothelial cell growth. Elevating GATA6-AS reduced TUG1 expression, but when TUG1 was overexpressed, it didn’t notably impact GATA6-AS levels. Increased GATA6-AS expression stimulated glioma cell proliferation while hindering apoptosis, whereas boosting TUG1 countered cell proliferation and mitigated the effects caused by increased GATA6-AS levels (171).

**Figure 7:**
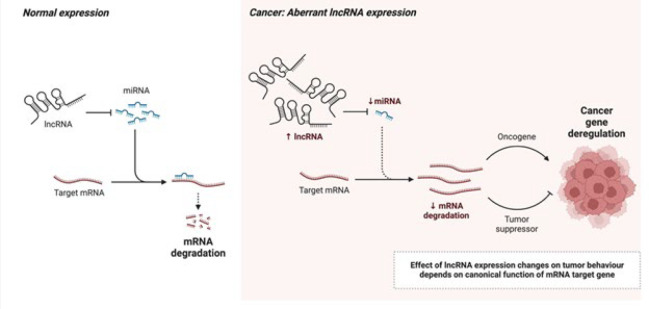
LncRNA and miRNA modulate cell death pathways in cancer, including apoptosis and autophagy

Moreover, a recently identified lncRNA, RP11-732M18.3, has been found to play a role in the growth of glioma by acting as an oncogene, interacting with 14-3-3β/α, which promotes the degradation of p21, ultimately leading to the proliferation of glioma cells and their transition through the G1/S cell cycle (172). Additionally, in an in vitro study, RP11-732M18.3 increased the levels of EP300, which led to VEGFA up-regulation, resulting in angiogenesis in glioma and, consequently, reduced survival rates in mice (173). Also, in glioma, the lncRNA RP5-833A20.1 restrained the proliferation of tumor cells, impeded cell cycle progression, and induced apoptosis by enhancing the expression of miR-382-5p, subsequently increasing the methylation level within the promoter region of nuclear factor IA, leading to the inhibition of its expression (174). The lncRNA and miRNA gene expression regulation in cancer is shown in Figure 7.

## Discussion

### Challenges and limitations of using ncRNAs as biomarkers or therapeutics in neurodegenerative diseases and brain cancer

The potential of ncRNAs as biomarkers and therapeutics in neurodegenerative diseases and brain cancer is increasingly recognized. Yet, their clinical application faces significant challenges due to the complexity of these disorders and the intricacies of the central nervous system (CNS)(175, 176). One of the fundamental obstacles lies in the stability and detection of ncRNAs in biological fluids, as these molecules are highly susceptible to degradation by nucleases. This instability complicates their reliable quantification, especially in cerebrospinal fluid (CSF) and blood (177), where ncRNA concentrations are often low. Moreover, variations in sample collection, processing, and analytical techniques introduce inconsistencies that hinder the reproducibility and standardization of ncRNA-based biomarkers, limiting their clinical utility in distinguishing between disease subtypes or progression stages. Another major limitation arises from the BBB, a highly selective structure that restricts the passage of molecules into the brain, posing a significant challenge for ncRNA-based therapeutics (178, 179). Effective delivery of therapeutic ncRNAs to target neurons or tumor cells requires advanced delivery platforms, such as lipid nanoparticles, exosomes, or viral vectors, which themselves introduce concerns regarding toxicity, immune activation, and unintended biodistribution. The heterogeneity of brain tumors, particularly glioblastoma, and the diverse pathophysiological mechanisms of neurodegenerative diseases further complicate the identification of universal ncRNA targets (180, 181), as different patient populations may exhibit varying ncRNA expression patterns. This variability affects diagnostic accuracy and raises concerns about the efficacy of ncRNA-based therapeutics across different individuals and disease stages. Additionally, the complex regulatory roles of ncRNAs present another layer of difficulty in their therapeutic application. Many ncRNAs function within intricate gene regulatory networks, where a single ncRNA can influence multiple signaling pathways. This pleiotropic nature increases the risk of unintended gene modulation, potentially leading to off-target effects that could exacerbate disease progression or introduce unforeseen side effects (182). In neurodegenerative diseases such as Alzheimer’s and Parkinson’s, where multiple molecular mechanisms contribute to neuronal degeneration, the challenge lies in ensuring that ncRNA-based interventions precisely modulate pathogenic pathways without disrupting essential physiological functions. Similarly, in brain cancer, where ncRNAs may act as oncogenes or tumor suppressors depending on the cellular context, fine-tuning ncRNA therapies is crucial to prevent undesired pro-tumorigenic effects (183, 184).

Another significant barrier to the clinical translation of ncRNA-based strategies is the need for extensive validation and large-scale clinical trials. While preclinical studies have demonstrated promising roles for ncRNAs in neurodegeneration and brain cancer, the lack of standardized protocols and limited reproducibility across independent studies hinders regulatory approval. Furthermore, ethical and regulatory challenges associated with RNA-based therapeutics necessitate rigorous safety evaluations, especially given concerns over long-term effects, immune responses, and potential unintended genetic alterations (185, 186). The high costs associated with developing and optimizing ncRNA-based diagnostic and therapeutic tools further limit their widespread adoption, particularly in low-resource settings where access to advanced molecular diagnostics and RNA-based therapies may be restricted (187). In conclusion, while ncRNAs offer a promising avenue for advancing diagnostics and therapeutics in neurodegenerative diseases and brain cancer, several critical challenges must be addressed before they can be integrated into clinical practice (188, 189). Issues related to stability, detection, reproducibility in biomarker applications and delivery, as well as specificity and regulatory complexity in therapeutics, remain significant obstacles. Overcoming these limitations requires continued advancements in RNA stabilization technologies, targeted delivery systems, and large-scale validation studies to ensure both safety and efficacy. Addressing these challenges will be essential for harnessing the full potential of ncRNAs in improving patient outcomes in these devastating neurological disorders (190, 191). 

### Therapeutic potential of ncRNAs: Clinical trials, delivery challenges, and overcoming barriers

The therapeutic potential of ncRNAs in neurodegenerative diseases and brain cancer is increasingly being explored, with several ongoing clinical trials investigating their efficacy (192). For instance, miRNAs such as miR-21 and miR-124 are being evaluated as therapeutic targets in glioblastoma (193), as they play crucial roles in tumor progression and immune modulation. In neurodegenerative diseases, miRNA-based therapies are being investigated for their ability to modulate key pathological processes, such as tau phosphorylation in Alzheimer’s disease or α-synuclein aggregation in Parkinson’s disease (194, 195). Some experimental RNA-based therapies, including small interfering RNAs (siRNAs) and antisense oligonucleotides (ASOs), have already reached clinical trials, such as tofersen (196), an ASO targeting SOD1 mutations in amyotrophic lateral sclerosis (ALS), highlighting the translational potential of ncRNA-based interventions. However, one of the major barriers to effective ncRNA therapy is the delivery challenge, particularly due to the BBB, which prevents most macromolecules from reaching the CNS. Traditional systemic delivery methods often result in rapid degradation of ncRNAs or off-target effects, limiting therapeutic efficiency. To overcome these challenges, several innovative delivery strategies have been developed (197). Nanoparticle-based carriers, such as lipid nanoparticles (LNPs), have shown promise in protecting ncRNAs from degradation while facilitating BBB penetration (198). Exosomes, naturally occurring extracellular vesicles, have also emerged as promising delivery vehicles due to their ability to cross the BBB and deliver functional ncRNAs to target cells (198, 199). Additionally, conjugation strategies, such as linking ncRNAs to cell-penetrating peptides or receptor-targeting ligands, are being explored to enhance specificity and uptake (200). While these advancements offer promising solutions, further research is needed to optimize delivery methods, improve tissue specificity, and ensure long-term safety, paving the way for ncRNA-based therapies to become viable treatments for neurodegenerative diseases and brain cancer (201).

### Insights for future research: Extracellular vesicles enriched in specific ncRNA

Secreted extracellular vesicles (EVs) have been postulated as a promising substitute for cell therapies. EVs comprise three categories: exosomes, microvesicles (MVs), and apoptotic bodies. The first two are being studied for their invaluable therapeutic and diagnostic roles in CNS disorders.

Because EVs’ cargo represents cellular status, the detection of neuron-originated EVs in the blood and CSF has progressed the field of CNS markers, providing a novel approach that allows the non-invasive assessment of CNS neurophysio-pathological state. Currently, the selective, reliable, and high-yield isolation of EVs from blood is still a difficult task, and there are no standardized methods for the special extraction of EVs (202, 203). As a good representation of cell status, the ncRNA profiles of plasma EVs are changed in neurodegenerative and CNS cancer conditions (204). For instance, in neuron-originated EVs isolated from AD cases, altered expression profiles of multiple miRs have been detected (205). Yet, there is only sporadic evidence quantifying lncRNAs in EVs in AD patients. Exosome lncRNA BACE1-AS is up-regulated in AD (206). Moreover, PCA3 and RP11-462G22.1 are overexpressed in EVs extracted from the AD cases’ CSF (207). Of note, PCA3 participates in developing prostate malignancy (208), while RP11-462G22.1 was initially detected as a muscular dystrophy-related lncRNA (209). However, their biological actions in AD are still not known. Further, plasma exosomal BACE1-AS, 51A, BC200, and BACE1 expression were quantified, and results showed that solely BACE1-AS was dysregulated when comparing AD specimens to healthy controls (210), indicating BACE1-AS as a potential marker in combination with cortical thickness (211). The lncRNAs PCA3 and RP11-462G22.1 have been implicated in various conditions beyond AD, and their expression profiles may not be entirely specific to AD. PCA3, for instance, is widely known for its association with prostate cancer, where it serves as a biomarker for cancer detection and progression. In contrast, RP11-462G22.1 has been suggested to play a role in various cellular processes, including those related to stress responses and cancer progression.

It is crucial to determine whether these lncRNAs’ expression is indeed specific to AD or if they are broader markers of cellular stress or pathological states that extend beyond AD (212). Some studies suggest that while the expression of specific lncRNAs may be up-regulated in neurodegenerative diseases like AD, they might also be involved in general cellular responses to stress, inflammation, or injury, rather than being exclusive to AD pathology. It would be helpful to clarify their role in AD, comparing their expression profiles in AD-specific contexts with those in other neurodegenerative diseases or stress-induced conditions. Such studies could involve comparing the levels of these lncRNAs in different cell types, tissue samples, or animal models, and examining how their expression correlates with AD-specific pathological features, such as amyloid plaque formation, tau tangles, and neuroinflammation. Additionally, understanding the underlying molecular pathways influenced by these lncRNAs could shed light on whether they are accurate biomarkers of AD or simply indicators of more generalized cellular responses. Therefore, further research is needed to determine whether PCA3 and RP11-462G22.1 are indeed specific biomarkers of AD or if their up-regulation is part of a broader cellular stress response that could be observed in various pathological conditions.

An important event of neurodegeneration is neuronal cell death. An instance in the case of PD is a particular neuron-specific miR-128 to be meaningfully attenuated in the patient-originated exosomes. Intriguingly, a concomitant attenuated expression of miR-128 was found in the cell models of PD. Bhattacharyya and colleagues showed that up-regulation of miR-128 could avoid 6-OHDA-regulated mitochondrial superoxide generation and triggering of nerve cells’ death. This neuroprotective influence was shown to be triggered through miR-128-modulated suppression of FoxO3a activation, a transcriptional element of apoptosis. miR-128 up-regulation led to lowered pro-apoptotic FoxO3a targets, Fas ligand (FasL), and PUMA (213). FasL is an apoptotic factor that could also exert hyperinflammatory effects under special conditions. Additional downstream miR-128 up-regulation suppressed the triggering of caspases-8/9/3, avoiding both the intrinsic and extrinsic apoptosis processes. In addition, up-regulation of the miR-128 avoided reduction of synaptic proteins- Synaptophysin and PSD-95, and decreased neurite shortening, as a result, maintaining overall neuronal integrity. As a result, our research shows the intracellular role of miR-128 in neuronal cell death and CNS degeneration and its implications as a marker being detectable in the circulating exosomes of PD cases (214). These concepts have also been applied to other neurodegenerative disorders, which is beyond the scope of this review. However, the lesson learned is that ncRNA delivery through different body organs could be made possible through EVs, which could be engineered (215) or through stimulation of cells, and be produced with up/down-regulated amounts of certain ncRNA content for therapeutic purposes.

While there are many benefits to using cell-free treatments, more research is required to establish EV-derived ncRNAs as novel biomarkers of neurodegenerative disease development and progression. Also, while their therapeutic targeting has been attempted in clinical trials of various neurological disorders, such trials are somewhat lacking in the field of neurodegenerative disorders. EV engineering to enhance specific targeting is also an avenue that deserves more attention in the future. To make considerable advancements that shift clinical practice and borders in neurodegeneration science and omics data, taking into account a wide range of ncRNAs at once by novel computational approaches may hold the key to success.

The isolation of EVs is a critical step in ncRNA analysis, as the method used can significantly influence the quality, quantity, and integrity of the EVs, thus impacting the overall results of the analysis. Different EV isolation methods vary in their ability to yield pure, intact vesicles while minimizing contamination with other cellular components. These variations can lead to discrepancies in ncRNA profiles, affecting reproducibility and the interpretation of results. Common EV isolation techniques include ultracentrifugation, density gradient centrifugation, precipitation-based methods, size-exclusion chromatography, and immunoaffinity capture (216). Ultracentrifugation, one of the most widely used methods, involves high-speed centrifugation to pellet EVs based on size and density. However, this method can co-isolate other particles such as protein aggregates or lipoproteins, leading to contamination that can affect the purity and integrity of the extracted ncRNAs. Furthermore, the high forces involved may also cause vesicle rupture, which could alter the composition of the ncRNA cargo (217). Density gradient centrifugation offers a more refined approach, allowing EVs to be separated from other contaminants based on their buoyant density. While this method improves the purity of isolated EVs, it is time-consuming and requires sophisticated equipment (218). Precipitation-based methods, such as those using commercial reagents, are often faster and easier to perform but may result in lower purity due to the co-precipitation of non-EV components. Size-exclusion chromatography (SEC) provides an alternative approach by separating particles based on size, offering a more straightforward, scalable solution (219). However, SEC may still fail to completely exclude small contaminating molecules, particularly in samples with low EV concentrations. Immunoaffinity capture, which uses antibodies targeting specific surface markers of EVs, can provide highly specific isolation of a subpopulation of EVs. However, this technique is limited by the availability and specificity of antibodies, and it may not capture all EV subtypes present in a sample. In addition, antibody efficiency variations can affect the reproducibility of the results. The method chosen for EV isolation can profoundly affect the resulting ncRNA profiles (220). For instance, different isolation methods may lead to variability in the amount and type of ncRNAs, such as miRNAs, lncRNAs, or circular RNAs, detected within the EVs. These variations could be attributed to differences in vesicle size distribution, purity, or RNA extraction efficiency. Furthermore, contamination with non-vesicular RNA or cellular debris may result in biased ncRNA profiles, complicating downstream analyses like quantitative PCR, next-generation sequencing, or microarray-based profiling (220).

To ensure reproducibility and reliability in ncRNA analysis, it is essential to standardize EV isolation protocols and carefully select the most appropriate method based on the specific research question and the intended application. Researchers should also report detailed methodologies, including the isolation techniques and their associated limitations, to allow for better comparison and validation of results. Ultimately, optimizing EV isolation protocols and minimizing the impact of isolation-related biases will be critical in achieving consistent and reproducible ncRNA profiles, which is necessary for advancing the therapeutic potential of EV-based ncRNA delivery systems (221).

EVs for ncRNA-based therapy present several technical challenges that must be addressed to maximize the therapeutic potential of EVs. One of the primary challenges lies in optimizing the loading efficiency of ncRNAs into EVs. Achieving high loading efficiency while preserving the stability and function of the ncRNAs within the vesicles is a significant hurdle. Techniques such as electroporation, sonication, and chemical transfection have been used, but they often result in low efficiency or unintended damage to the cargo or EV structure. Developing more efficient methods, such as utilizing specific targeting peptides or proteins, could potentially enhance the selective incorporation of ncRNAs into EVs (222).

Additionally, ensuring that ncRNAs are selectively packaged into EVs, instead of being randomly incorporated with other cellular contents, remains a critical challenge. The sorting of cargo into EVs is controlled by cellular machinery, and alterations in these pathways may lead to reduced specificity and efficacy. Strategies to optimize selective ncRNA packaging into EVs could involve engineering the vesicle-producing cells or modifying EV surface proteins to interact with specific cellular components involved in RNA packaging. Omics technologies, including transcriptomics and proteomics, offer the potential to identify the molecular pathways and factors involved in this sorting process, thus providing insights into how selective cargo packaging can be enhanced (223).

Scalability and reproducibility of engineered EV production are also significant challenges in the field. Large-scale production of EVs, suitable for clinical use, remains a bottleneck due to the complexity and cost of existing methods for isolation and purification. Furthermore, maintaining consistent quality and purity across different batches of EVs is crucial for therapeutic applications. Advances in bioreactor systems, coupled with omics technologies, such as metabolomics and proteomics, could offer solutions to these challenges. By analyzing the molecular profiles of EVs at various stages of production, it may be possible to establish standardized protocols that ensure high-yield, high-quality EV production on a larger scale (224).

The immunogenicity of engineered EVs is another area that requires careful consideration. While EVs are naturally occurring vesicles that generally exhibit low immunogenicity, the process of engineering them for therapeutic use may alter their surface characteristics, making them susceptible to immune system recognition. Surface modifications, such as the addition of PEGylation or targeting ligands, are being explored to address this. Omics approaches, including immune profiling via transcriptomics and proteomics, can be used to monitor immune responses to engineered EVs *in vivo*, helping to identify and mitigate potential immunogenicity issues.

Finally, achieving effective *in vivo* targeting and delivery of engineered EVs to specific tissues or cells is another major hurdle. Despite the promising ability of EVs to cross biological barriers such as the BBB, their distribution and accumulation in the desired target tissues remain unpredictable. Engineering EVs with specific surface markers or targeting peptides can improve tissue-specific delivery. Omics technologies, such as single-cell RNA sequencing and proteomics, can be employed to track the biodistribution of EVs and evaluate their interactions with various cell types *in vivo*, thereby improving the precision and efficacy of ncRNA-based therapies.

In conclusion, while EVs offer significant promise as vehicles for ncRNA-based therapy, several technical challenges must be addressed to optimize their use in clinical settings. Integrating omics approaches such as transcriptomics, proteomics, and metabolomics into the design and production of engineered EVs holds the potential to provide valuable insights that will help overcome these challenges and enable the successful translation of EV-based therapies into clinical practice.

The DNA in the nucleus is transcribed by RNA polymerase into pre-mRNA, which undergoes processing to form mature mRNA. This mRNA is then translated by ribosomes into proteins. Simultaneously, ncRNAs like small nuclear RNA (snRNA), small nucleolar RNA (snoRNA), and long non-coding RNA (lncRNA) play regulatory roles. Additionally, ncRNAs lead to the formation of other RNA molecules, such as circular RNA (circRNA), transfer RNA (tRNA), and small interfering RNA (siRNA). These molecules are involved in various cellular processes, including gene regulation, protein synthesis, and pseudogene formation

**Table 1:** Role of non-coding RNA in neurodegenerative diseases

Non-Coding RNA	Role in neurodegenerative diseases	Associated diseases	Mechanism/Function	Reference
LncRNAs	Regulate gene expression, protein folding, and cellular processes	Alzheimer’s Disease (AD), Parkinson’s Disease (PD), Huntington’s Disease (HD)	Influence amyloid plaque formation, tau aggregation, and autophagic pathways. Some, like MALAT1, have neuroprotective roles	(28, 29)
miRNAs (small ncRNAs)	Modulate gene expression by binding to mRNA, controlling protein synthesis	AD, PD, HD, frontotemporal dementia (FTD)	Regulate tau phosphorylation, Aβ formation, and synaptic function. Specific miRNAs like miR-9, miR-101, and miR-16 are implicated in AD	
piRNAs	Protect the genome by silencing transposons and controlling gene expression	Neurodegenerative diseases associated with genomic instability	Suppress transposons and stabilize genome integrity, potentially affecting neurodegenerative mechanisms	
Vault RNAs	Contribute to cell survival, response to stress, and regulation of apoptosis	AD, PD, Amyotrophic Lateral Sclerosis (ALS)	Modulate cellular responses to stress, apoptosis, and inflammatory processes	

**Table 2:** The role of miRNA in neurodegenerative disease

Disease	miRNA name	PMID	Description
Parkinson’s disease	miR-let-7d	29082467	Findings strongly suggest that let-7d is essential in DA neuronal cell injury
	miR-126	24559646	Levels of miR-126 may play a functional role in DA neurons and PD pathogenesis by down-regulating IGF-1/PI3K/AKT signaling, and its inhibition could be a mechanism of neuroprotection
	miR-16-1	25054189	This study revealed a novel regulatory mechanism of Hsp70 expression, which might contribute to the development of PD
	miR-205	23125283	Findings suggest that down-regulation of miR-205 may contribute to the potential pathogenic elevation of LRRK2 protein in the brains of patients with sporadic PD. At the same time, overexpression of miR-205 may provide an applicable therapeutic strategy to suppress the abnormal up-regulation of LRRK2 protein in PD
	miR-214	26349993	The loss of miR-214 in PD resulted in the increase of α-synuclein expression, which was the potential mechanism underlying the neuroprotective effects of Resveratrol
	miR-22	27631550	Analysis showed that miR-22 mediated 6-OHDA-induced PC12 cell survival and proliferation by targeting TRPM7. Taken together, the present study showed that miR-22 overexpression exhibited neuroprotective effects
	miR-221	29405726	MiR-221 plays a protective role in Parkinson's Disease via regulating PC12 cell viability and apoptosis by targeting PTEN. Therefore, miR-221 may serve as a potential therapeutic target for Parkinson's disease treatment
	miR-30e	29274035	These findings suggest that miR30e may be a key inflammation-mediated molecule that could be a potential target for PD therapeutics
	miR-34b	24892887	miR-34b levels are also significantly reduced in the putamen of incidental PD cases and along disease progression. Given that 3'UTR of A2AR contains a predicted target site for miR-34b, the potential role of this miRNA in protein A2AR levels was assessed
	miR-7	27003614	study indicated that miR-7 protects from MPP(+)-induced cell apoptosis in SH-SY5Y by directly targeting KLF4
	miR-7	27158385	The altered molecular expressions downstream of Bax and Sirt2 are also involved in miR-7 regulation of the MPP(+)-triggered neuronal apoptosis. These findings have implications for the potential application of miR-7 in PD treatment
Multiple sclerosis	miR-132	25136908	Over-expression of miR-132 in normal B cells significantly enhanced their production of lymphotoxin and tumor necrosis factor 伪. The over-expression of miR-132 also suppressed the miR-132 target, sirtuin-1
	miR-155	23818336	Results demonstrate that miR-155 regulates proinflammatory responses in both blood-derived and central nervous system (CNS)-resident myeloid cells and impacts subsequent adaptive immune responses. Differential miRNA expression may, therefore, provide insight into mechanisms responsible for distinct phenotypic and functional properties of myeloid cells, thus impacting their ability to influence CNS injury and repair
	miR-15b	28228555	Found that *O*-linked *N*-acetylglucosamine transferase is a potential target of miR-15b, enabling it to affect the transcriptional regulation of retinoic acid-related orphan receptor γT through *O*-linked *N*-acetylglucosamine glycosylation of NF-κB. These results contribute to the importance of miR-15b in Th17 differentiation and the pathogenesis of MS
	miR-17	24644282	T cell-specific miR-17-92 deficiency reduced TH17 differentiation and ameliorated experimental autoimmune encephalomyelitis (EAE) symptoms
	miR-223	27083389	miR223 promotes EAE, probably by enhancing DC activation and subsequently differentiating naive T cells toward Th1 and Th17 effector cells
	miR-326	29181619	MicroRNA-326 contributes to autoimmune thyroiditis by targeting the Ets-1 protein
	miR-448	28342869	MicroRNA-448 promotes multiple sclerosis development through induction of the Th17 response by targeting protein tyrosine phosphatase non-receptor type 2 (PTPN2)
Alzheimer’s Disease	miR-101	20395292	miR-101 is a negative regulator of APP expression and affects the accumulation of Abeta, suggesting a possible role for miR-101 in neuropathological conditions
	miR-101	28202389	Targeting the HDAC2/HNF-4A/miR-101b/AMPK Pathway Rescues Tauopathy and Dendritic Abnormalities in Alzheimer's Disease
	miR-124	26592243	Study indicates that miR-124 plays neuroprotective roles in AD Drosophila by targeting Delta in the Notch signaling pathway, which helps further our understanding of miRNAs in the molecular pathology of AD
	miR-124	26984601	Data suggest that miR-124, as an endogenous regulator of BACE1 protein, may play a role in AD onset induced by CCH
	miR-124-1, miR-124-2, miR-124-3	22178568	The miR-124 regulates the expression of BACE1/-secretase correlated with cell death in Alzheimer's disease
	miR-125b	29901156	Findings suggested that miR125b may regulate AD, neuronal cell growth, and apoptosis, via the regulation of inflammatory factors and oxidative stress by SphK1; therefore, miR125b may be involved in AD development
	miR-128	30328325	MiR-128 inhibitor decreased Aβ-mediated cytotoxicity by up-regulating PPAR-γ via inactivation of NF-κB in MCN and N2a cells, providing a new potential target in AD treatment
	miR-132	26362250	These findings support a role for miR-132/212 in regulating tau pathology in mice and humans and provide new alternatives for therapeutic development
	miR-139	28218780	Findings demonstrate that miR-139 exerts a pathogenic effect in AD by modulating CB2-mediated neuroinflammatory processes
	miR-146a	27241555	Overexpression of miRNA-146a in SH-SY5Y cells significantly decreased Lrp2 expression, resulting in a reduction of Akt activation and induction of proapoptotic caspase-3, thereby increasing cell apoptosis
	miR-146a	18801740	Data indicate that NF-kappaB-sensitive miRNA-146a-mediated modulation of CFH gene expression may in part regulate an inflammatory response in AD brain and in stressed HN cell models of AD, and illustrate the potential for anti-miRNAs as an effective therapeutic strategy against pathogenic inflammatory signaling.
	miR-16	26592823	Overexpression and inhibition of miR-16 in the cellular AD model with primary hippocampal neurons decreased and increased apoptosis
	miR-188	25378159	miR-188-3p expression was up-regulated by 2-AG or peroxisome proliferator-activated receptor-γ (PPARγ) agonists and suppressed by PPARγ antagonism or NF-κB activation
	miR-206	27277332	The role of miR-206 was determined by gain and loss of function experiments in LPS-treated microglia. The results demonstrated that miR206 up-regulation enhanced LPSinduced inflammation and Aβ release in microglia
	miR-212	26362250	Treatment of AD mice with miR-132 mimics restored memory function and tau metabolism in part. Finally, miR-132 and miR-212 levels correlated with insoluble tau and human cognitive impairment. These findings support a role for miR-132/212 in regulating tau pathology in mice and humans and provide new alternatives for therapeutic development
	miR-29a, miR-29b-1	18434550	miR-29a, -29b-1, and -9 can regulate BACE1 expression in vitro. The miR-29a/b-1 cluster was significantly (and AD-dementia-specific) decreased in AD patients displaying abnormally high BACE1 protein
	miR-29c	26212654	miR-29c directly mediated down-regulation of NAV3 protein expression in vitro. The mouse NAV3 mRNA has a functional miR-29c binding site in the 3' UTR, which is localized between 830-836 bp of the 3'UTR region
	miR-29c	25955795	The results demonstrated that the up-regulation of miR-29c promoted learning and memory behaviors in SAMP8 mice, at least partially, by increasing the activity of protein kinase A/cAMP response element-binding protein, involved in neuroprotection. This evidence suggested that miR-29c may be a promising potential therapeutic target against AD
	miR-324	29956723	miR3243p may be potential biomarkers and therapeutic targets for AD
	miR-33	26538644	Demonstrate that inhibition of microRNA-33 increases lipidation of brain ApoE and reduces Aβ levels by inducing ABCA1. We provide a unique approach for AD therapeutics to increase ApoE lipidation and reduce Aβ levels via pharmacological inhibition of microRNA *in vivo*
	miR-339	24352696	miR-339-5p levels were significantly reduced in brain specimens isolated from AD patients compared to age-matched controls. Therefore, miR-339-5p regulates BACE1 expression in human brain cells and is most likely dysregulated in at least a subset of AD patients, making this miRNA a novel drug target
	miR-34a	26459758	Significant decrease in miR-34a and TAp73 was observed in the cortex of a transgenic (Tg) mouse model of AD, which correlated well with cell cycle reentry observed in the neurons of these animals. Significantly, the overexpression of TAp73α and miR-34a reversed cell cycle-related neuronal apoptosis (CRNA)
	miR-34a	27235866	Results raise the possibility that pathophysiology-induced activation of a specific transcription factor may lead to increased expression of the miR-34a gene, and miR-34a-mediated concurrent repression of its target genes in neural networks may result in dysfunction of synaptic plasticity, energy metabolism, and resting state network activity
	miR-34c	26402112	miR-34c blockade up-regulated VAMP2 expression and rescued synaptic failure as well as learning and memory deficits caused by Aβ
	miR-922	24950120	miR-922 increasing the levels of phosphorylated tau by regulating UCHL1 levels, contributed to the pathogenesis of AD. Our study partly explained one of the mechanisms underlying the down-regulation of UCHL1 levels in AD patients and could enrich the tau pathology's content in AD's pathogenesis
	miR-937	26316079	Overexpression of as-miR-937 in MSCs may substantially improve the therapeutic effects of MSCs on AD, possibly through augmenting Brn-4 levels in MSCs
	miR-98	27541017	Findings demonstrate that miR-98-5p modulates SNX6 expression and thus plays a critical role in the accumulation of Aβ. Therefore, miR-98-5p may be a novel therapeutic target for AD
Huntington disease	miR-125b-1, miR-125b-2, miR-146a, miR-150, miR-214	22048026	Showed that miR-214, miR-150, miR-146a, and miR-125b targeted HTT. Besides, the exogenous expression of wild-type miRNAs reduced HTT aggregates formed by the recombinant exon1 of the HTT gene
	miR-214	26307536	Showed that increased expression of miR-214 observed in the HD cell model could target MFN2, alter mitochondrial morphology, and deregulate the cell cycle. Inhibition of miR-214 could be a possible intervention target in HD pathogenesis
	miR-22	23349832	Data show that miR-22 has multipartite anti-neurodegenerative activities, including the inhibition of apoptosis and the targeting of mRNAs implicated in the etiology of HD. These results motivate additional studies to evaluate the feasibility and therapeutic efficacy of manipulating miR-22 *in vivo*
	miR-124	26109954	Findings suggest that microRNA-124 slows the progression of Huntington’s disease, possibly through its important role in neuronal differentiation and survival
	miR-22	23349832	Show that miR-22 has multipartite anti-neurodegenerative activities, including the inhibition of apoptosis and the targeting of mRNAs implicated in the etiology of HD
Amyotrophic lateral sclerosis	miR-125b	26794445	By restoring A20 levels, miR-125b inhibition then sustains motor neuron survival
	miR-206	24664281	In mutant mice lacking miR-206, reinnervation is impaired following nerve injury, and loss of NMJs is accelerated in a mouse model of amyotrophic lateral sclerosis (ALS)
	miR-155	25381879	Overexpression of miR-155 in the SOD1 mouse and both sporadic and familial human ALS. Targeting miR-155 in SOD1 mice restores dysfunctional microglia and ameliorates disease
	miR-132-5p, miR-132-3p, miR-124-3p, and miR-133a-3p	37189452	Highlighted the potential of miR-132-5p, miR-132-3p, miR-124-3p, and miR-133a-3p expression levels in plasma as biomarkers of preclinical progression for G376D-TARDBP-associated ALS
	miR-335-5p	32152380	The down-regulation of miR-335-5p, which has an effect on mitophagy, autophagy, and apoptosis in SH-SY5Y neuronal cells, could have a role in the motor neuron loss observed in ALS
	miR-7-2-3p, miR-26a-1-3p, miR-224-5p and miR-206	37531027	Found that miR-7-2-3p, miR-26a-1-3p, miR-224-5p, and miR-206 are good study candidates to understand the pathophysiology of ALS

Each panel represents distinct disease clusters, connected by lines to central nodes indicating shared molecular mechanisms or genetic similarities. (A) Diseases related to the central nervous system, muscular, and autoimmune disorders, highlighting connections such as Alzheimer’s disease, Parkinson’s disease, and multiple sclerosis. (B) Cluster showing associations with chronic obstructive pulmonary disease, liver neoplasms, and myeloid leukemia, indicating links with respiratory, liver, and blood-related pathologies. (C) Grouping around lymphoproliferative disorders and autoimmune diseases, such as lymphoma, multiple sclerosis, and rheumatoid arthritis. (D) Panel showing connections between respiratory infections, skin diseases, and neurological disorders, including respiratory syncytial virus, psoriasis, and amyotrophic lateral sclerosis. (E) Disease associations centered around systemic infections, autoimmune disorders, and neurodegenerative conditions, such as tuberculosis, sepsis, and Huntington disease

The non-coding RNAs that are encapsulated within exosomes have been identified as playing crucial and multifaceted roles in the complex biological processes that govern the proliferation and invasion of glioma cells, as well as in the promotion of angiogenesis, the establishment of an immunosuppressive microenvironment, and the development of resistance to various therapeutic interventions aimed at treating this aggressive form of brain cancer (140)(Produced by Biorender.com with permission from Biorender)

Comparison of the regulation of target mRNA under normal conditions versus cancerous states due to changes in lncRNA expression. On the left, in a typical scenario, miRNA levels allow miRNA to bind to target mRNA, leading to mRNA degradation, thus maintaining normal gene expression. On the right, in cancer, there is an abnormal increase in lncRNA and a decrease in miRNA. This imbalance reduces the degradation of target mRNA, leading to the overexpression of oncogenes or underexpression of tumor suppressors. Such dysregulation contributes to cancer progression and tumor formation. The caption highlights that the impact of lncRNA expression changes on tumor behavior depends on the target gene’s role in cancer biology

## Conclusion

The role of ncRNAs in neurodegeneration and brain tumors is undeniably significant, as they mediate gene regulation and orchestrate key pathological events such as abnormal protein aggregation, apoptosis, and immune system dysregulation. These mechanisms provide promising targets for therapeutic intervention. However, despite the considerable progress made in understanding the functions of ncRNAs, several challenges remain that hinder their translation into clinical applications. One of the primary challenges lies in isolating and characterizing EVs, which are crucial carriers of ncRNAs. Current methods for EV isolation, such as ultracentrifugation, density gradient centrifugation, and immunoaffinity capture, vary in efficiency, purity, and reproducibility. These inconsistencies can lead to discrepancies in ncRNA profiles, complicating the interpretation of results and limiting the reliability of EVs as diagnostic or therapeutic tools. Therefore, there is an urgent need for standardized protocols that ensure high-yield, high-quality EV isolation, minimizing contamination and preserving the integrity of ncRNA cargo. Additionally, the clinical application of ncRNA-based therapies faces significant hurdles, particularly in efficiently loading ncRNAs into EVs and their targeted delivery to specific tissues or cells. Techniques such as electroporation and chemical transfection often result in low efficiency or damage to the EV structure. Future research should focus on developing more efficient methods for ncRNA loading, such as utilizing specific targeting peptides or proteins, and optimizing the selective packaging of ncRNAs into EVs. Furthermore, the scalability and reproducibility of engineered EV production must be addressed to meet the demands of clinical use. Another critical area for future research is the clinical validation of ncRNA biomarkers. While numerous ncRNAs have been identified as potential diagnostic and prognostic markers for neurodegenerative diseases and brain tumors, their specificity and sensitivity need to be rigorously validated in large-scale clinical studies. Preclinical testing of EV-based therapies, including their biodistribution, pharmacokinetics, and immunogenicity, is also essential to ensure their safety and efficacy before transitioning to human trials. In conclusion, while ncRNAs and EVs hold immense potential as diagnostic tools and therapeutic agents, significant challenges remain in their isolation, characterization, and clinical application. Future research should prioritize the development of standardized protocols for EV isolation, the optimization of ncRNA loading and delivery, and the clinical validation of ncRNA biomarkers. By addressing these challenges, we can pave the way for the successful translation of ncRNA-based therapies into clinical practice, ultimately improving outcomes for patients with neurodegenerative diseases and brain tumors.

## Declaration

We acknowledge the use of ChatGPT to improve the writing of introduction and abstract.
